# Novel Cyclic Tetrapeptides as Neuraminidase Inhibitors from a Sponge-Associated *Penicillium* sp. SCSIO41035

**DOI:** 10.3390/md23100377

**Published:** 2025-09-26

**Authors:** Weihao Chen, Xiangliu Chen, Mengjing Cong, Jianglian She, Xiaoyan Pang, Shengrong Liao, Bin Yang, Xuefeng Zhou, Yonghong Liu, Fuquan Xu, Junfeng Wang

**Affiliations:** 1 State Key Laboratory of Tropical Oceanography, Guangdong Key Laboratory of Marine Materia Medica, South China Sea Institute of Oceanology, Chinese Academy of Sciences, Guangzhou 510301, China; chenweihao17@mails.ucas.ac.cn (W.C.); chenxiangliu23@mails.ucas.ac.cn (X.C.); c3021632921@163.com (M.C.); shejianglian20@mails.ucas.ac.cn (J.S.); xypang@scsio.ac.cn (X.P.); ljrss@126.com (S.L.); bingo525@163.com (B.Y.); xfzhou@scsio.ac.cn (X.Z.); 2 University of Chinese Academy of Sciences, 19 Yuquan Road, Beijing 100049, China; 3 Sanya Institute of Ocean Eco-Environmental Engineering, Sanya 572000, China; 4 Jiangsu Key Laboratory of Marine Biotechnology, Jiangsu Ocean University, Lianyungang 222000, China

**Keywords:** cyclic peptide, spiroquinazoline, sponge-associated fungus, neuraminidase inhibitor, theoretical calculation

## Abstract

Four new compounds and three new natural products (**1**−**7**), including three novel cyclic tetrapeptides (penicopeptides B−D), along with three known spiroquinazoline analogs (**8**–**10**), were isolated from rice medium cultures of a sponge-associated *Penicillium* sp. SCSIO41035. The structural elucidations, including the determination of absolute configurations, were accomplished by comprehensive analyses utilizing NMR spectroscopy, HRESIMS, optical rotation data, X-ray crystallography experiments and electronic circular dichroism calculations. Differential NMR signals between symmetric units in cyclotetrapeptides **1** and **2** arise from the asymmetric solution conformations as investigated through conformational searching and theoretical calculations. The asymmetric conformations were primarily caused by the flexibility of the tyrosine residue’s phenyl side chain, with its substantial electron density significantly influencing the NMR signals of nearby groups. Bioactivity screening results displayed that isolated compounds demonstrated good neuraminidase inhibitory activity, with inhibition rates ranging from 43.16% to 85.40% at a concentration of 100 µg/mL.

## 1. Introduction

Cyclic tetrapeptides are a class of natural products with significant potential in drug discovery, characterized by their unique structural features and diverse biological activities [1,2]. Their cyclic structure confers stability and contributes to their potency, while their low molecular weight and favorable lipophilicity enhance drug-like properties. According to Lipinski’s rule of five, cyclic tetrapeptides typically exhibit manageable hydrophobicity and an appropriate balance of hydrogen-bond donors and acceptors, facilitating cellular uptake and solubility [3]. The presence of hydrogen-bond acceptors and donors contributes significantly to the drug-like properties of cyclic tetrapeptides, enabling their diverse biological activities such as potent inhibition of histone deacetylase, broad-spectrum antimicrobial activity, efficacy against dinoflagellates, and cytotoxic effects on various cell lines, etc. [4,5]. Cyclic tetrapeptides as medium-sized peptides, often derived from microorganism sources, contain amino acids such as leucine, isoleucine, phenylalanine, valine, and proline [4]. Endolides A and B, from a sponge-derived fungus *Stachylidium* sp. were found to be vasopressin and serotonin-receptor interacting *N*-methylated peptides [6]. Asperterrestide A, a novel cyclic tetrapeptide isolated from the marine fungus *Aspergillus terreus* SCSGAF0162 showed low IC_50_ values (6.2 and 6.4 μM) against two human carcinoma cell lines and also inhibited the influenza strains H1N1 (15 μM) and H3N2 (8.1 μM) [7]. As for now, a natural cyclic tetrapeptide, romidepsin (Istodax), as an HDAC inhibitor, was approved by FDA in 2009 for the treatment of T-cell lymphoma [8].

The chemical synthesis of cyclic tetrapeptides remains one of the most formidable challenges in peptide chemistry, primarily due to the extreme ring strain inherent in the 12/13/14-membered macrocycle [4,9]. This strain arises from unfavorable transannular interactions and the high entropic cost of forcing the *N*- and *C*-termini into close proximity, a prerequisite for efficient head-to-tail cyclization. Consequently, even seemingly simple sequences often fail to cyclize or give poor yields, especially when composed of l-amino acids lacking turn-inducing elements [9,10]. Racemization at the *C*-terminal residue during activation is another persistent risk, particularly when *N*-methylated amino acids are involved [11]. Despite s tactics like the use of d-amino acids, or proline, *N*-methylation, and ring-contraction strategies and recent advances in coupling reagents, microwave-assisted cyclization, and transition-metal-mediated C–H activation, have been developed to lower the activation barrier, a universal protocol remains elusive and every cyclic tetrapeptides still requires case-by-case optimization [12,13,14].

**Figure 1:**
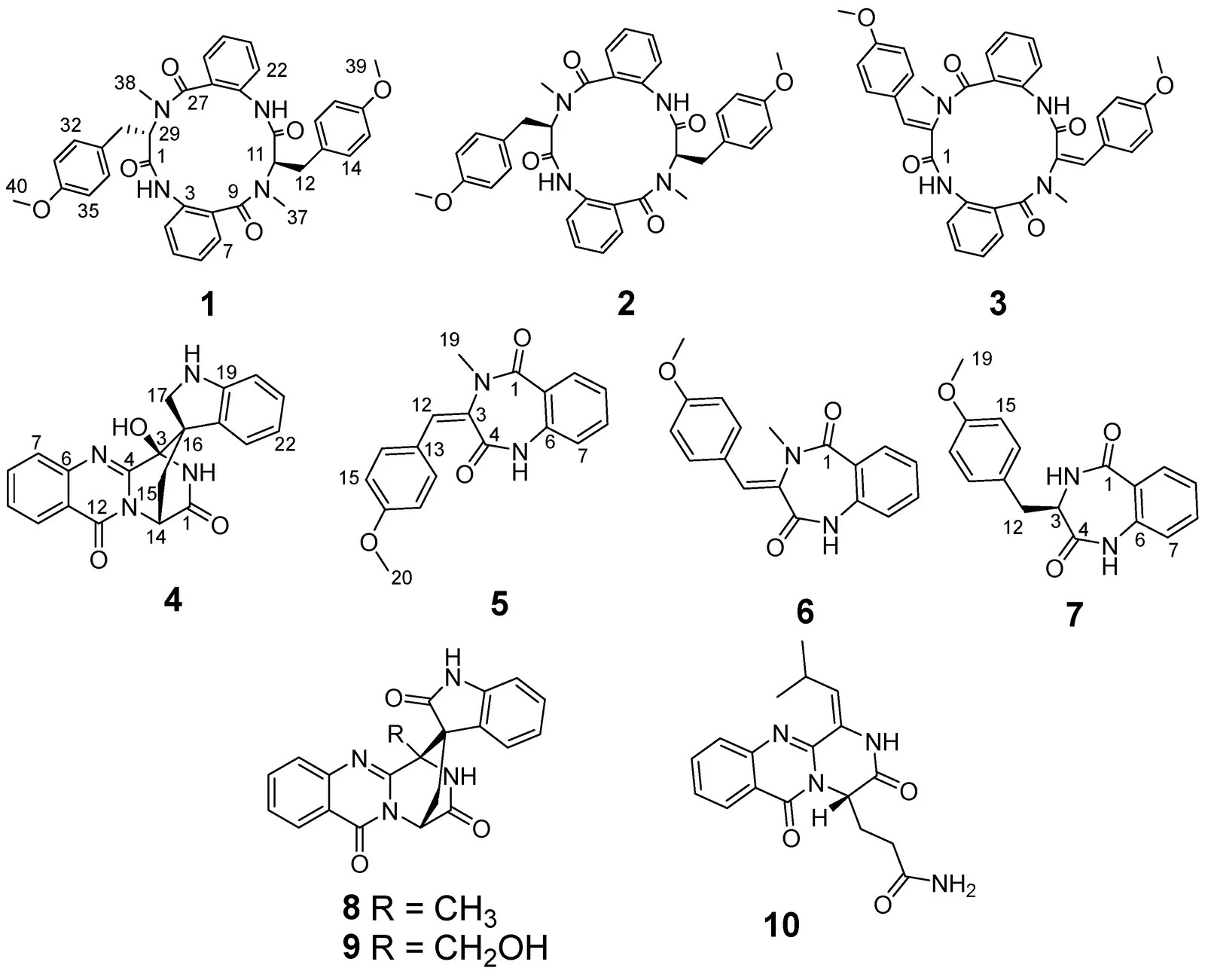
Structures of compounds **1**–**10**.

During our continuous exploration of cyclic peptide molecules from marine-derived fungi [15,16,17], a chemical investigation of a sponge-associated *Penicillium* sp. SCSIO41035 resulted in the isolation and identification of three novel cyclic tetrapeptides (**1**–**3**), one novel spiro-quinazolines (**4**), three cyclic dipeptide natural products (**5**–**7**), and three known spiro-quinazoline analogues (**8**–**10**), as shown in Figure 1. Bioactivity screening results showed compounds **1**–**10** exhibited varying levels of neuraminidase inhibitory activity. Herein, the details of the isolation, structural identification and biological activities of **1**–**10** are described.

## 2. Results and Discussion

**Figure 2:**
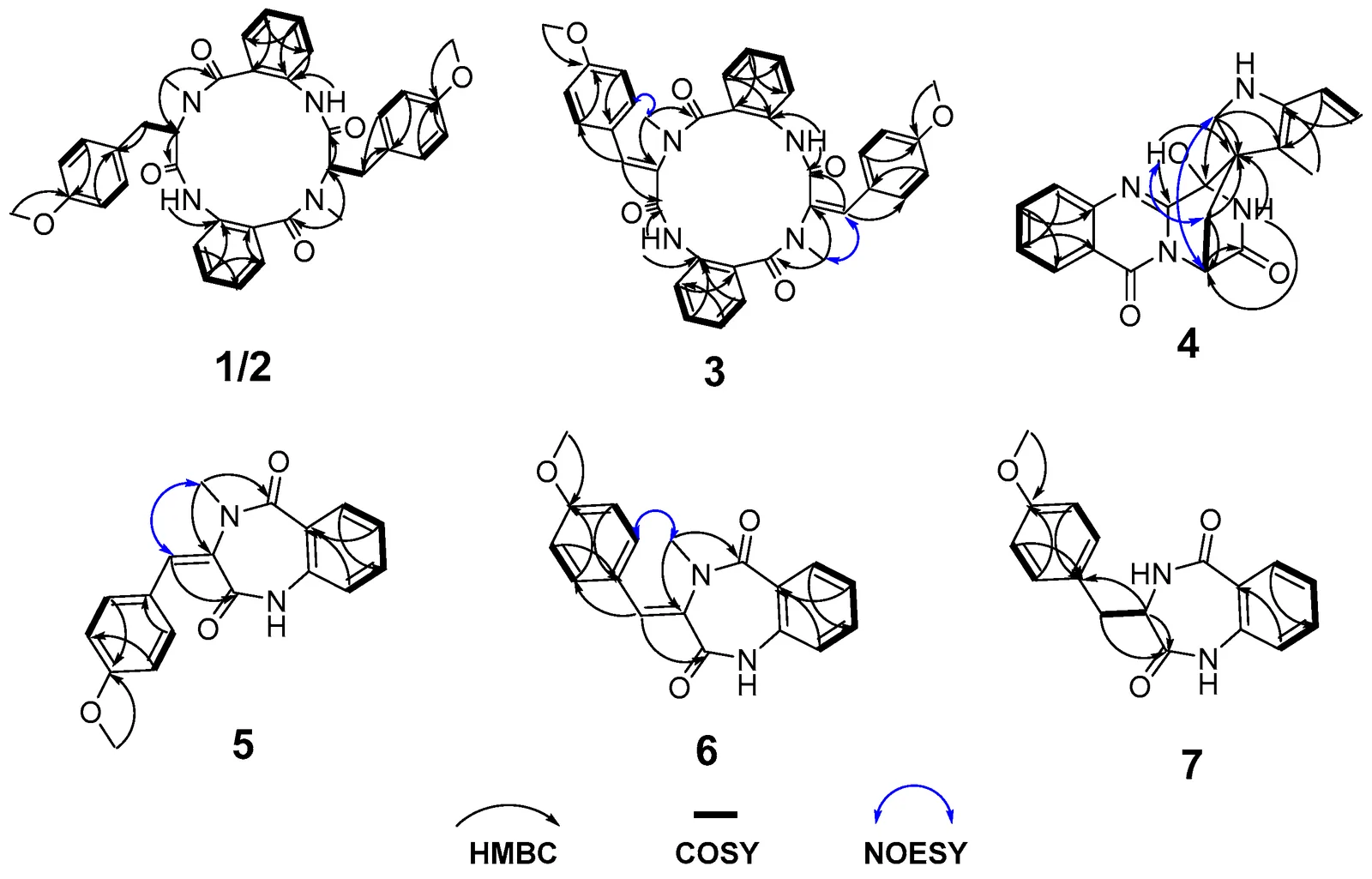
Key HMBC, COSY and NOESY correlations of compounds **1**−**7**.

Penicopeptide B (**1**) was obtained as a yellow oil and its molecular formula was established as C_36_H_36_N_4_O_6_ by the HR-ESI-MS data at *m/z*: 621.2711 ([M + H]^+^, calcd. for C_36_H_37_N_4_O_6_^+^, 621.2708). The ^1^H NMR data (Table 1) displayed two overlapped methoxy signals at *δ*_H_ 3.73 (6H, s, 39/40-OCH_3_), two amide *N*-methyl signals at *δ*_H_ 2.93 (3H, s, H-38) and *δ*_H_ 3.14 (3H, s, H-37), two amino proton signals at *δ*_H_ 9.60 (1H, s, NH-2) and 9.26 (1H, s, NH-20), and two amino acid protons at *δ*_H_ 4.28 (1H, dd, *J* = 8.5, 6.1 Hz, H-11) and *δ*_H_ 4.22 (1H, dd, *J* = 11.0, 6.3 Hz, H-29). The ^13^C NMR and DEPT spectra indicated that compound **1** possessed 36 carbon signals, involving two methoxyls, two *N*-methyls, two methylenes, eighteen methines and twelve quaternary carbons (Table 2). Analyses of the 2D NMR data indicated compound **1** was a cyclic tetrapeptide skeleton compound containing two tyrosine groups and two aminobenzoic acid moities, which was confirmed by HMBC signals (Figure 2) from NH-2 to C-1 (*δ*_C_ 172.0) and C-3 (*δ*_C_ 126.9), from H_3_-37 to C-9 (*δ*_C_ 168.5) and C-11 (*δ*_C_ 56.7), from H-11 to C-19 (*δ*_C_ 170.3), from NH-20 to C-19 and C-21 (*δ*_C_ 127.5), from H_3_-38 to C-27 (*δ*_C_ 166.1) and C-29 (*δ*_C_ 68.7). The cyclic tetrapeptide skeleton of **1** was identical to that of penicopeptide A by comparison of their ^1^H and ^13^C NMR data [18]. Compared with penicopeptide A, two additional methoxy groups at C-16 (*δ*_C_ 158.6) and C-36 (*δ*_C_ 158.8) in compound **1** were confirmed by HMBC signals from the overlapped methoxy hydrogen signals to C-16 and C-36, respectively. Thus, the planar structure of **1** was identified as a novel cyclic tetrapeptide composed of two units, each unit being formed by the condensation of *N*-methyl-7-methoxy-tyrosine with *O*-aminobenzoic acid (Figure 1).

Penicopeptide C (**2**) was obtained as a yellow oil and assigned the same molecular formula of C_36_H_36_N_4_O_6_ as **1** on the basis of its HRESIMS data. Both NMR data of **1** and **2** (Table 1 and Table 2) were collected in the same CDCl_3_ solvent and showed the same number of carbon atoms and chemical shifts in ^13^C NMR spectrum, but slight differences chemical shifts were observed in ^1^H NMR spectrum. Therefore, compound **2** was identified to possess the same cyclic tetrapeptide skeleton and might be an epimer of compound **1**. To further determine the absolute configuration, 0.6 mg of compounds **1** and **2** were dissolved in 1.0 mL of 6 N HCl and heated to 110 °C for 1 h. The hydrolysis products were dissolved in methanol and their specific rotation was measured. The structure of compound **1** was determined as a mesomere, containing two *N*-methyl-7-methoxy-tyrosines with opposite configurations by the specific optical rotation value of its hydrolysis product ([*α*${]}_{D}^{25}$= 0.001°, *c* 0.1, MeOH) [19], while compound **2** contained only L-*N*-methyl-7-methoxy-tyrosine residue as deduced by the specific optical rotation value of its hydrolysis product ([*α*${]}_{D}^{25}$= −21.1°, *c* 0.1, MeOH). Thus, the absolute configurations of compounds **1** and **2** were determined to be 11*R*, 29*S* and 11*R*, 29*R,* respectively.

**Figure 3:**
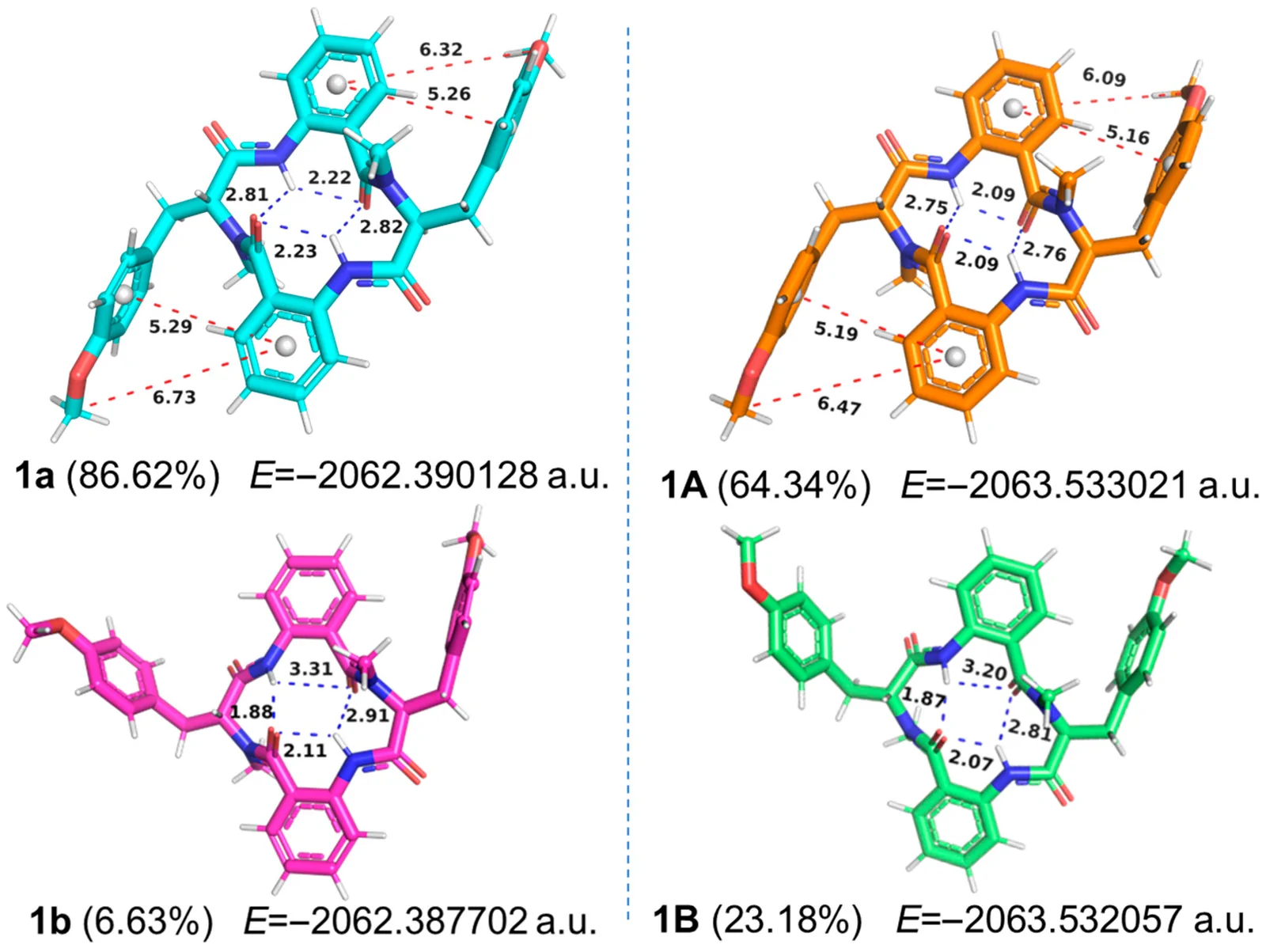
Conformational analysis of **1** in vacuum (**1a** and **1b**) and in solvent with SCRF chloroform solvation model (**1A** and **1B**) at B3LYP/6-31G* level of theory.

Theoretical analysis indicated that compounds **1** and **2**, as structurally symmetrical cyclic tetrapeptides, should exhibit overlapping NMR signals for their equivalent positions. However, experimental results reveal distinct chemical shift patterns for the two compounds. This apparent discrepancy may arise from asymmetrical conformational preferences adopted by these macrocyclic structures in solution-phase dynamics, as supported by comprehensive conformational analysis using molecular modeling. Initially, conformational search and energy minimization were conducted for the structures of compounds **1** and **2** in a vacuum environment, and subsequently performed the same procedures in chloroform solvent model (Supplementary Materials). The optimal conformation (**1a**) of **1** in vacuum accounted for 86.62% of the Boltzmann distribution. From the three-dimensional structure and the strength of the intramolecular hydrogen bonds (2.22, 2.23, 2.81, and 2.82 Å, blue dashed lines, Figure 3), the overall molecular framework appeared symmetrical. In contrast, within the solvation model, the proportion of the best conformation (**1A**) decreased to 64.34%, even though the intramolecular hydrogen bonds 2.09, 2.09, 2.75, and 2.76 Å still suggested that the overall framework maintained a symmetrical state. The above result indicated that the conformation of the three-dimensional structure of the cyclic tetrapeptide molecule was significantly affected under the solvation model, consistent with previous reports [4]. Additionally, through more detailed analysis, it was found that in both vacuum and solvation models, the distances (red dash lines) between adjacent phenyl rings (π-π interactions), as well as the distances (red dash lines) from the methoxy group to the center of the phenyl ring of the *o*-aminobenzoic acid within **1a** and **1A**, were not equal, which indicated that the overall structures of **1a** and **1A** were in an asymmetric state.

**Figure 4:**
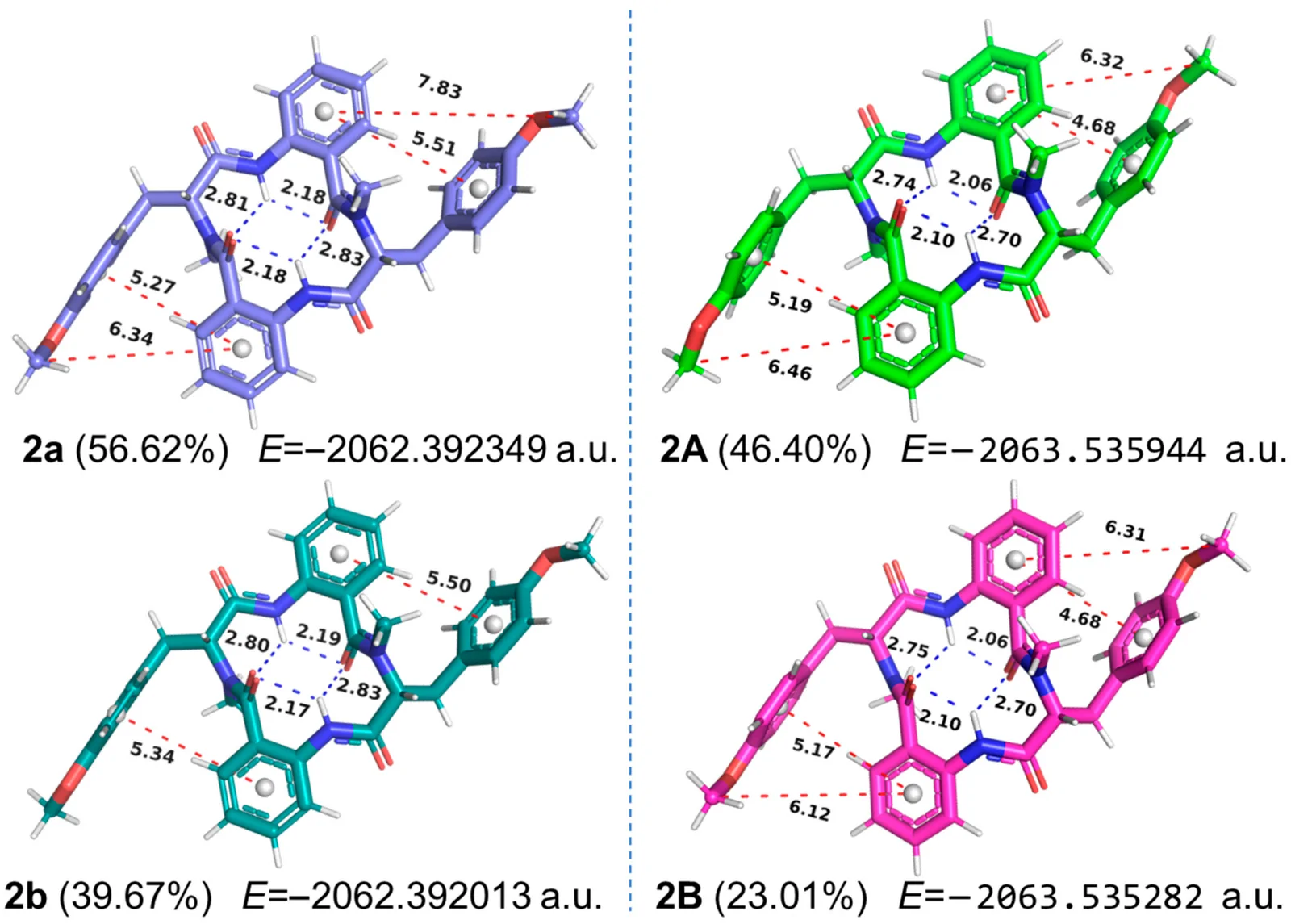
Conformational analysis of **2** in vacuum (**2a** and **2b**) and in solvent with SCRF chloroform solvation model (**2A** and **2B**) at B3LYP/6-31G* level of theory.

As for **2** (Figure 4), the proportion of the first two relatively symmetrical conformations decreased from 96.29% in vacuum (**2a** and **2b**) to 69.41% in the solvation mode (**2A** and **2B**). Meanwhile, due to the differences in the strength of intramolecular hydrogen bonds and the distances between adjacent phenyl rings, compound **2** was also in an asymmetric state in the solvation model, as the substantial electron density of tyrosine phenyl side chain significantly influenced the NMR signals of nearby groups. Thus, in chloroform solution, the conformational distributions of compounds **1** and **2** were significantly influenced by solvation effects and the inherent flexibility of the tyrosine’s aromatic side chains, resulting in an asymmetric molecular structure and giving rise to distinct NMR chemical shifts. The NMR-derived asymmetric characteristics of compounds **1** and **2** have also been reported in another cyclic tetrapeptide molecule, penicopeptide A [18], which was similarly investigated through molecular modeling, but without considering intramolecular hydrogen bonds and π-π interactions between phenyl rings.

Penicopeptide D (**3**) was also obtained as a white solid and assigned the molecular formula of C_36_H_32_N_4_O_6_ on the basis of its HRESIMS data ([M + H]^+^ *m*/*z* 617.2422, calcd for C_36_H_33_N_4_O_6_^+^, 617.2395). The cyclic tetrapeptide nature of **3** was inferred from the high similarity of its NMR data to that of compounds **1** and **2** (Table 1 and Table 2). The main difference was that compound **3** possessed two additional double bonds at *δ*_H/C_ 6.65 (s)/133.1 (CH-12); *δ*_C_ 130.6 (C-11) and *δ*_H/C_ 6.90 (s)/133.5 (CH-30); *δ*_C_ 131.1 (C-29) replacing two amino acid protons, as confirmed by HMBC signals from H_3_-37 (*δ*_H_ 3.48, s) to C-11, from H-12 to C-19 (*δ*_C_ 168.3) and C-14 (*δ*_C_ 130.7), from H_3_-38 (*δ*_H_ 3.22, s) to C-29, and from CH-30 to C-1 (*δ*_C_ 172.0) (Figure 2). The 11*E*, 29*Z* configurations of **3** were determined by NOESY correlations of H-12/H-37 and H3-38/H-32 (*δ*_H_ 7.32, d, *J* = 8.8 Hz), respectively. Thus, compound **3** was elucidated as a novel cyclic tetrapeptide containing two units that formed by the condensation of *N*-methyl-2-ene-7-methoxy-tyrosine with *o*-aminobenzoic acid.

**Figure 5:**
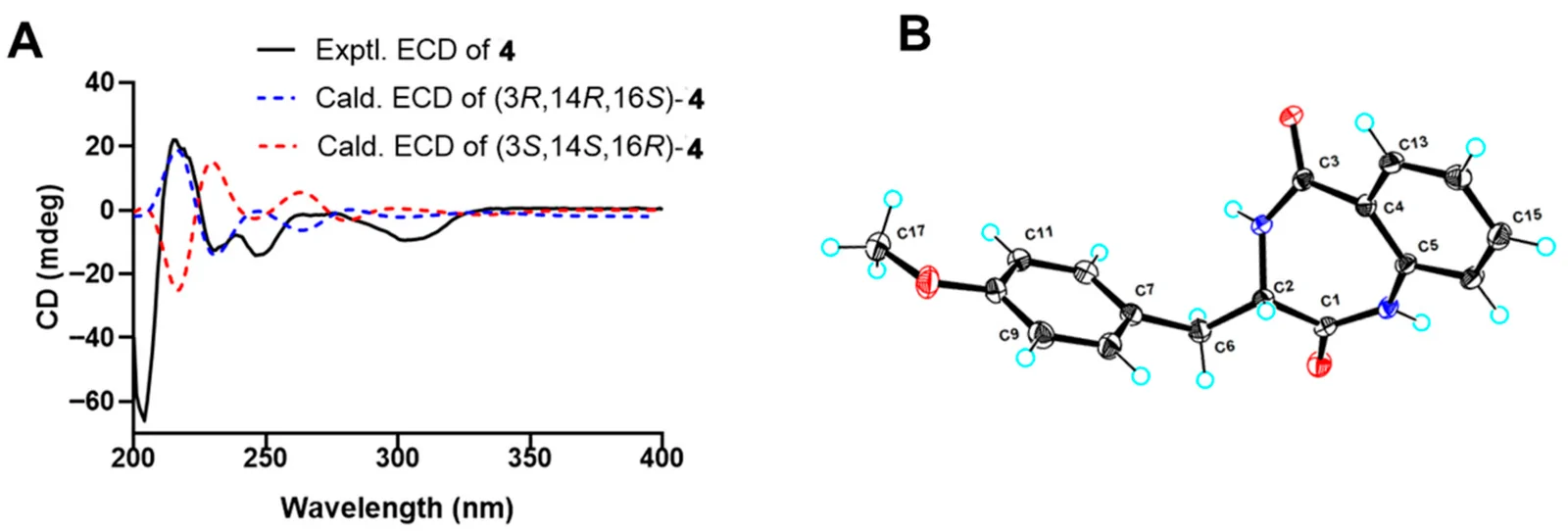
(**A**) Spectra for exptl. ECD of **4** and cald. ECD of (3*R*,14*R*,16*S*)- and (3*S*,14*S*,16*R*)-**4**. (**B**) ORTEP drawing of compound **7**.

The molecular formula of compound **4,** a white solid, was confirmed as C_20_H_16_N_4_O_3_ based on the HRESIMS analysis at *m/z* 361.1300 ([M + H]^+^, calcd. for C_20_H_17_N_4_O_3_^+^ 361.1295) indicating fifteen degrees of unsaturation. The 1D NMR spectra (Table 3 and Table 4) and ^1^H-^1^H COSY spectrum of **4** showed the presences of two 1,2-disubstituted benzene ring systems and a -*N*-CH-CH_2_- sequence, indicating a tryptophan residue as those in alantrypinone (**8**) [20], a spiroquinazoline from *Penicillium thymicola*. Compared to alantrypinone (**8**), the methyl group was replaced by a hydroxyl group (*δ*_H_ 6.31, d, *J* = 3.8 Hz, 3-OH) in **4**, as evidenced by the chemical shift in C-3 (*δ*_C_ 70.7) and the HMBC signals from 3-OH to C-4 (*δ*_C_ 153.8) and C-16 (*δ*_C_ 59.3). The carbonyl group in the indole ring of **8**, was reduced to a methylene group (*δ*_C/H_ 40.0/2.79, dd, *J* = 14.5, 1.9 Hz; 2.48, dd, *J* = 14.5, 4.3 Hz, CH_2_-17) of **4**, as confirmed by the HMBC signals from H_2_-17 to C-3 (*δ*_C_ 70.7), C-16 (*δ*_C_ 59.3) and C-24 (*δ*_C_ 123.7) (Figure 2). Thus, the planar structure of **4** was determined as shown and named as penicopeptide E The relative configuration of **4** was determined as 3R*, 14R*, 16S* based on the NOESY correlations between 3-OH and H_2_-15 (*δ*_H_ 4.07, d, *J* = 12.6 Hz; 3.58, dd, *J* = 12.6, 3.8 Hz), as well as between H-14 (*δ*_H_ 5.40, m) and H_2_-17. Two possible diastereoisomers, (3*R*,14*R*,16*S*)-**4** and (3*S*,14*S*,16*R*)-**4**, were then subjected to time-dependent density functional theory (TDDFT) ECD calculations. The absolute configuration of **4** was determined as 3*R*,14*R*,16*S*, as the experimental curve coincided with the calculated curve of (3*R*,14*R*,16*S*)-**4** (Figure 5A).

Penicopeptides F and G (**5** and **6**) were obtained as white solid and assigned the same molecular formula of C_18_H_16_N_2_O_3_ based on their HRESIMS data at *m*/*z* 309.1233 and 309.1240 ([M + H]^+^, calcd for C_18_H_17_N_2_O_3_^+^, 309.1234), respectively. At the same time, their almost identical NMR data (Table 3 and Table 4) displayed the feature of a *ortho*-disubstituted and a *para*-disubstituted benzene ring systems at *δ*_C/H_ 120.7/6.97 (d, *J* = 8.0 Hz, CH-7), 132.4/7.43 (td, *J* = 7.7, 1.6 Hz, CH-8), 125.6/7.25 (overlapped, CH-9), 131.8/7.94 (dd, *J* = 8.0, 1.6 Hz, CH-10), 130.8/7.34 (d, *J* = 8.8 Hz, 2CH-14/18), and 114.3/6.83 (d, *J* = 8.8 Hz, 2CH-15/17) for **5**, while at *δ*_C/H_ 120.4/6.94 (d, *J* = 8.0 Hz, CH-7), 132.8/7.45 (td, *J* = 8.0, 1.6 Hz, CH-8), 125.2/7.24 (t, *J* = 7.6 Hz, CH-9), 131.6/8.00 (dd, *J* = 8.0, 1.6 Hz, CH-10), 131.5/7.33 (d, *J* = 8.8 Hz, 2CH-14/18), and 114.6/6.83 (d, *J* = 8.8 Hz, 2CH-15/17) for **6**. The cyclic dipeptide skeleton of **5** and **6** composed of *N*-methyl-2-ene-7-methoxy-tyrosine and *o*-aminobenzoic acid was determined by HMBC signals from *N*-CH_3_-19 to C-1 and C-3, and from the olefinic proton H-12 (*δ*_H_ 6.65, s, in **5**; 7.79, s, in **6**) to C-4 (Figure 1). The 3*E* configuration of **5** and 3*Z* configuration of **6** were confirmed by NOESY correlations between H-12 and H_3_-19 (*δ*_H_ 3.49, d, *J* =3.2 Hz) of **5**, and between H-14/18 and H3-19 (*δ*_H_ 3.22, s) of **6**. Compounds **5** and **6**, characterized as dehydro-4-methoxycyclopeptines, have been previously reported as synthetic intermediates of cyclopeptin [21] and as substrates for elucidating the mechanism of oxoglutarate-dependent oxygenase [22]. This is the first report of compounds **5** and **6** being isolated as new natural products from a sponge-associated *Penicillium* sp. SCSIO41035.

Compound **7** was assigned the molecular formula of C_17_H_16_N_2_O_3_ based on the HRESIMS analysis at *m*/*z* 297.1235 ([M + H]^+^, calcd. for C_17_H_17_N_2_O_3_^+^, 297.1234). Analysis of the NMR data (Table 3 and Table 4) revealed that **7** was a cyclic dipeptide analogue closely related to **6**, with the structural difference being the absences of an *N*-methyl group and an olefinic moiety in **7**. The amino acid proton at *δ*_H_ 4.00 (1H, dt, *J* = 8.4, 6.1 Hz, H-3) and a methylene signal *δ*_C/H_ 33.7/3.01 (dd, *J* =14.6, 8.4 Hz, H-12a); 3.35 (dd, *J* =14.6, 6.1 Hz, H-12b) were observed in 1D NMR data. The 3*R* configuration of **7** was confirmed by the single-crystal diffraction result (Figure 5B, CCDC number 2453921). Thus, compound **7** was determined as a cyclic dipeptide composed of a l-7-methoxy-tyrosine and *o*-aminobenzoic acid. Compound **7,** named penicopeptide H, together with its crystal structure, was first reported as a natural product from a sponge-associated *Penicillium* sp. SCSIO41035. It has also been previously described as a substrate for investigating the non-enzymatic rearrangement synthesis of 4′-methoxyviridicatin [22].

The other known compounds were identified as spiroquinazoline analogs alantrypinone (**8**) [20], serantrypinone (**9**) [23], and aurantiomide C (**10**) [24], respectively, by comparisons of NMR and MS data with literature data. The isolated compounds were evaluated for antiviral effects against HSV-1/2, dengue, and Zika viruses and antitumor effects against cancer cell lines PC-3, 22Rv1, and A549. However, all tested compounds were inactive to both viruses and cancer cell lines at concentrations of 20.0 μM. All these isolates were also tested for their enzyme inhibitory activity against pancreatic lipase, acetylcholinesterase, and neuraminidase. The results showed that under a concentration of 100 µg/mL, ten compounds only demonstrated good neuraminidase inhibitory activity, with inhibition rates ranging from 43.16% to 85.40% (Table 5), while the inhibition rates for the other two enzymes were both below 35%. Regarding structure-activity relationship, cyclic dipeptides (63.65–85.40%) generally showed superior inhibitory activity compared to cyclic tetrapeptides (43.16–58.67%). Additionally, the presence of the double bond at C-3 further enhanced the neuraminidase activity, as demonstrated by the following comparisons: 58.67% for **3** vs 43.16% for **2** and 85.40% for **5** vs 63.65% for **6**.

## 3. Conclusions

In summary, the rice medium cultures of a sponge-associated *Penicillium* sp. SCSIO41035 were investigated and resulted in the isolation and identification of four new compounds and three new natural products (**1**−**7**), including three novel cyclic tetrapeptides (penicopeptides B−D) and three known spiroquinazoline analogs (**8**–**10**). The structural elucidations, including the determination of absolute configurations, were achieved through comprehensive analyses utilizing NMR spectroscopy, HRESIMS, optical rotation data, X-ray crystallography, and electronic circular dichroism calculations. Notably, differential NMR signals between symmetric units in cyclotetrapeptides **1** and **2** were attributed to asymmetric solution conformations, as elucidated through conformational searching and theoretical calculations. These asymmetric conformations were primarily caused by the flexibility of the tyrosine residue’s phenyl side chain, with its substantial electron density significantly influencing the NMR signals of nearby groups. Bioactivity screening revealed that the isolated compounds demonstrated good neuraminidase inhibitory activity, with inhibition rates ranging from 43.16% to 85.40% at a concentration of 100 µg/mL. These findings highlighted the potential of these compounds as inhibitors of neuraminidase.

## 4. Materials and Methods

### 4.1. General Experimental Procedures

Optical rotations were measured in a PerkinElmer MPC 500 (Waltham, MA, USA) polarimeter at 25 °C. UV spectra were recorded in MeOH using Shimadzu UV-2600 spectrophotometer (Shimadzu, Kyoto, Japan). ECD data were acquired by the Chirascan circular dichroism spectrometer (Applied Photophysics, Leatherhead Surrey, UK). Crystallographic data were collected on an XtaLAB AFC12 (RINC): a Kappa single diffractometer using Cu Kα radiation. NMR spectra were obtained at 500 MHz using a Bruker Avance spectrometer (Bruker, Billerica, MA, USA). HRESIMS spectra were generated on a Bruker miXis TOF-QII mass spectrometer (Bruker, Billerica, MA, USA). Thin-layer chromatography (TLC) and column chromatography (CC) were performed using silica gel GF254 (10–40 µm) and silica gel (200–300 mesh) from Qingdao Marine Chemical Factory (Qingdao, China). TLC spots were visualized under 254 nm UV light. Semipreparative high-performance liquid chromatography (HPLC) was conducted using an ODS column (NanoChrom, ChromCore 120-C18, 10 × 250 mm, 5 µm).

### 4.2. Fungal Material

The fungal strain SCSIO41035 was isolated from an unidentified sponge species collected at Zhongsha Islands in the South China Sea. The ITS gene region (ITS1-5.8S-ITS2) of strain SCSIO41035 was amplified by PCR. DNA sequencing showed it shared a significant homology (99.24%) to the sequence of *Penicillium albocoremium* IBT 10682 (accession No. NR_138271.1), so it was designated as *Penicillium* sp. and named as *Penicillium* sp. SCSIO41035. The producing strain was stored on MB agar (malt extract 15 g, artificial sea salt 10 g, agar 16 g, H_2_O 1 L, pH 7.4−7.8) slants at 4 °C and deposited in the CAS Key Laboratory of Tropical Marine Bioresources and Ecology, South China Sea Institute of Oceanology, Chinese Academy of Sciences, Guangzhou, China.

### 4.3. Fermentation and Extraction

The seed culture was prepared by inoculating spores of strain SCSIO41035 into three 150 mL flasks, each containing 30 mL seed medium (malt extract: 15 g, sea salt: 2.5 g, distilled water: 1 L and pH: 7.4–7.8), and incubated at 25 °C on a rotary shaker (178 rpm) for 3 days. The seed culture was then transferred into 1 L × 36 conical flasks with solid rice medium (each flask contained 200 g rice, 3 g sea salt and 200 mL naturally sourced water), and the large-scale fermentation of the strain was carried out at 25 °C for 30 days. The total rice culture was crushed and extracted with EtOAc for five times to yield 64.3 g crude gum.

### 4.4. Isolation and Purification

The EtOAc extract was subjected to vacuum liquid chromatography on a silica gel column using step gradient elution with MeOH−CH_2_Cl_2_ (0−100%) to separate into six fractions based on TLC properties. Fraction 2 was divided into three parts (Frs 2-1–2-3) followed by a Sephadex LH-20 column eluted with MeOH. Fr. 2-1 was purified by semipreparative HPLC (65% MeOH−H_2_O, 2 mL/min) to yield **1** (4.1 mg, *t*_R_ 20.5 min), **2** (3.8 mg, *t*_R_ 22.6 min), and **6** (8.2 mg, *t*_R_ 13.9 min). Fr. 2-2 was separated by semipreparative HPLC (75% MeOH–H_2_O with 0.1% TFA, 2 mL/min) to afford **3** (3.3 mg, *t*_R_ 18.3 min), **9** (6.1 mg, *t*_R_ 23.5 min), and **10** (5.0 mg, *t*_R_ 26.2 min). Fraction 3 was divided into four parts (Frs 3-1−3-4) followed by a Sephadex LH-20 column eluted with MeOH. Fr. 3-2 was purified by semipreparative HPLC (60% MeOH−H_2_O, 2 mL/min) to yield **4** (3.3 mg, *t*_R_ 14.5 min) and **5** (2.9 mg, *t*_R_ 16.3 min). and **6** (5.2 mg, *t*_R_ 13.9 min). Fraction 5 was also subjected to semi-preparative HPLC eluting with 58% CH_3_OH/H_2_O (0.1% TFA) to give **7** (3.2 mg, *t*_R_ 15.5 min, 2 mL/min) and a subfraction 5-2. Compound **8** (10.5 mg, *t*_R_ 18.5 min) was obtained from the subfraction 5-2 by semi-preparative HPLC eluting with 54% CH_3_OH/H_2_O (2 mL/min).

*Penicopeptide B* (**1**): a yellow oil, [*α*${]}_{D}^{25}$ = −2.41 (*c* 0.1, MeOH); UV (MeOH) *λ*_max_ (log *ε*) 215 (3.98), 227.6 (3.90), 283 (3.85) nm; ECD (*c* 0.40 mM, MeOH) *λ* (*Δε*) 215 (0.77), 217 (0.69), 231 (−2.44), 243 (0.18), 246 (0.17), 255 (0.67), 282 (−0.48) nm; ^1^H NMR (CDCl_3_, 500 MHz) and ^13^C NMR (CDCl_3_, 125 MHz) NMR data, see Table 1 and Table 2; HRESIMS *m*/*z* 621.2711 [M + H]^+^ (calcd. for C_36_H_37_N_4_O_6_, 621.2708), 643.2517 [M + Na]^+^ (calcd. for C_36_H_36_N_4_NaO_6_, 643.2527).

*Penicopeptide C* (**2**): a yellow oil, [*α*${]}_{D}^{25}$ = −38.7 (*c* 0.1, MeOH); UV (MeOH) *λ*_max_ (log *ε*) 213 (3.99), 283 (3.06) nm; ECD (*c* 0.48 mM, MeOH) *λ* (*Δε*) 221 (−2.79), 235 (−14.56), 246 (0.17), 258 (4.21), 286 (−8.55) nm; ^1^H NMR (CDCl_3_, 500 MHz) and ^13^C NMR (CDCl_3_, 125 MHz) NMR data, see Table 1 and Table 2; HRESIMS *m*/*z* 621.2711 [M + H]^+^ (calcd. for C_36_H_37_N_4_O_6_, 621.2708), 643.2517 [M + Na]^+^ (calcd. for C_36_H_36_N_4_NaO_6_, 643.2527).

*Penicopeptide D* (**3**); a white solid, UV(MeOH) *λ*_max_ (log *ε*) 205.0 (3.78), 210.0 (3.78), 281.8 (3.17) nm; ^1^H NMR (CDCl_3_, 500 MHz) and ^13^C NMR (CDCl_3_, 125 MHz) NMR data, see Table 1 and Table 2; HRESIMS *m*/*z* 617.2422 [M + H]^+^ (calcd. for C_36_H_33_N_4_O_6_, 617.2395), 639.2233 [M + Na]^+^ (calcd. for C_36_H_32_N_4_NaO_6_, 639.2214).

*Penicopeptide E* (**4**): a white solid, [*α*${]}_{D}^{25}$ = 42.45 (*c* 0.1, MeOH), UV (MeOH) *λ*_max_ (log *ε*) 210 (3.88), 228 (3.05), 266 (3.07), 277 (3.08), 305 (2.97), 316 (2.82) nm; ECD (*c* 0.45 mM, MeOH) *λ* (*Δε*) 215 (+21.96), 230 (−12.67), 246 (−14.20), 275 (−0.98), 305 (−9.32) nm; ^1^H NMR (DMSO-*d*_6_, 500 MHz) and ^13^C NMR (DMSO-*d*_6_, 125 MHz) NMR data, see Table 3 and Table 4; HRESIMS *m/z*: 361.1300 [M + H]^+^ (calcd. for C_20_H_17_N_4_O_3_: 361.1295), 383.1111 [M + Na]^+^ (calcd. for C_20_H_16_N_4_NaO_3_: 383.1115), 721.2531 [2M + H]^+^ (calcd. for C_40_H_33_N_8_O_6_: 721.2518), 743.2321 [2M + Na]^+^ (calcd. for C_40_H_33_N_8_NaO_6_: 743.2337).

*Penicopeptide F* (**5**): a white solid, UV(MeOH) *λ*_max_ (log *ε*) 213.6 (4.50), 286.6 (4.17) nm; ^1^H NMR (CDCl_3_, 500 MHz) and ^13^C NMR (CDCl_3_, 125 MHz) NMR data, see Table 3 and Table 4; HRESIMS *m*/*z* 309.1240 [M + H]^+^ (calcd. for C_18_H_17_N_2_O_3_, 309.1234), 331.1060 [M + Na]^+^ (calcd. for C_18_H_16_N_2_NaO_3_, 331.1053).

*Penicopeptide G* (**6**): a white solid, UV(MeOH) *λ*_max_ (log *ε*) 212.6 (4.67), 294.4 (4.35) nm;^1^H NMR (CDCl_3_, 500 MHz) and ^13^C NMR (CDCl_3_, 125 MHz) NMR data, see Table 3 and Table 4; HRESIMS *m*/*z* 309.1233 [M + H]^+^ (calcd. for C_18_H_17_N_2_O_3_, 309.1234), 331.1050 [M + Na]^+^ (calcd. for C_18_H_16_N_2_NaO_3_, 331.1053).

*Penicopeptide H* (**7**), a light-yellow oil, [*α*${]}_{D}^{25}$ = 6.19 (*c* 0.1, MeOH); UV(MeOH) *λ*_max_ (log *ε*) 216 (3.88), 283 (2.68) nm; ECD (c 0.35 mM, MeOH) *λ* (*Δε*) 212 (+14.72), 231 (–16.46), 250 (+32.57), 292 (−3.57) nm; ^1^H NMR (CDCl_3_, 500 MHz) and ^13^C NMR (CDCl_3_, 125 MHz) NMR data, see Table 3 and Table 4; HRESIMS *m*/*z* 297.1235 [M + H]^+^ (calcd. for C_17_H_17_N_2_O_3_, 297.1234), 319.1072 [M + Na]^+^ (calcd. for C_17_H_17_N_2_NaO_3_, 319.1053), 593.2380 [2M + H]^+^ (calcd. for C_34_H_33_N_4_O_6_, 593.2395), 615.2156 [2M + Na]^+^ (calcd. for C_34_H_33_N_4_NaO_6_, 615.2214).

### 4.5. Crystallographic Data for Penicopeptide H (***7***)

Moiety formula: C_17_H_18_N_2_O_4_ (M = 314.33 g/mol), monoclinic, Crystal size = 0.13 × 0.04 × 0.04 mm^3^, Space group = P2_1_; unit cell dimensions: *a* = 7.03720(10) Å, *b* = 8.6825(2) Å, *c* = 12.8359(3) Å, *V* = 754.45(3) Å^3^, *ρ*_calcd_ = 1.384 g/cm^3^, Z = 2, T = 100 (10) K, *μ* (CuK_α_) = 0.822 mm^−1^, A total of 7956 reflections were measured (7.16° ≤ 2Θ ≤148.314°) with 2928 independent reflections (*R*_int_ = 0.0306, *R*_sigma_ = 0.0334). Final *R* indexes [*I* ≥ 2*σ* (*I*)]: *R*_1_ = 0.0303, *wR*_2_ = 0.0745. Final *R* indexes [all data]: *R*_1_ = 0.0334, *wR*_2_ = 0.0759. Largest diff. peak and hole = 0.18 and −0.18 eÅ^−3^. Goodness-of-fit on F^2^ = 1.063. Flack parameter = −0.04(10). Crystallographic data for structure **7** have been deposited with the Cambridge Crystallographic Data Centre as supplementary publication number CCDC 2453921. Copies of the data can be obtained, free of charge, on application to CCDC, 12 Union Road, Cambridge CB21EZ, UK [fax: +44(0)-1223-336033 or e-mail: deposit@ccdc.cam.ac.uk].

### 4.6. Conformational Analyses of ***1*** and ***2***

The conformational searches for compounds **1** and **2** were carried out by the Confab programs in OpenBabel software (version 3-1-1) [25], which can systematically generate diverse low-energy conformations that are proposed to be close to crystal structures. The parameters were set as follows: RMSD cutoff = 2, Energy cutoff = 30, Conformer cutoff = 100,000. The generated conformers were subjected to preliminary optimization at the semi-empirical PM7 level using the MOPAC software (version 2016), which was invoked by the Molclus program [26]. The conformers with a Boltzmann population of over 1% were reoptimized using density functional theory (DFT) at the B3LYP/6-31G* (GD3BJ) level under vacuum or solvent condition using the ORCA 5.0.3 program [27].

### 4.7. ECD Calculation of Compound ***4***

The preliminary conformational distribution search was also performed using the Confab algorithm in OpenBabel software. Due to the absence of flexible side chains, only one conformation per configuration was reoptimized using density functional theory (DFT) at the B97-3c level under vacuum condition using the ORCA 5.0.3 program [27]. Subsequently, frequency calculations were performed following geometry optimization to verify that all the structures correspond to energy minima and have no imaginary frequency. The overall theoretical calculation of ECD was conducted in MeOH using time dependent density functional theory (TD-DFT) at the PBE0/def2-TZVP level for the optimized conformers. Rotatory strengths for a total of 30 excited states were calculated. The ECD spectra of different conformers were generated using the Multiwfn program [28] with a half-bandwidth of 0.3–0.4 eV, according to the Boltzmann calculated contribution of each conformer. The calculated ECD spectrum was corrected for offset based on the difference between the calculated and experimental UV spectra.

### 4.8. Neuraminidase Inhibitory Assay

The neuraminidase inhibitory activity was evaluated using a Neuraminidase In-hibitor Screening Kit (Beyotime Institute of Biotechnology, Shanghai, China). Assays were carried out in 96-well plates. For each well, 70 μL of neuraminidase assay buffer, 10 μL of neuraminidase solution, and 10 μL of test compound (dissolved in DMSO; final con-centration 100 μg/mL) were gently mixed. The reaction was initiated by adding 10 μL of the fluorogenic substrate to each well, and the plate was incubated at 37 °C for 30 min. Fluorescence was measured on an EnSpire microplate reader with excitation at 322 nm and emission at 450 nm. The fluorescence intensity of neuraminidase was measured at several concentrations to generate a standard curve relating fluorescence signal to enzyme concentration. The fluorescence corresponding to the highest neuraminidase concentration was set as 0% inhibition. Each sample well initially contained neuraminidase at this concentration, and the residual neuraminidase concentration after the reaction was determined from the measured fluorescence using the standard-curve equation. The inhibition percentages were then calculated. Oseltamivir acid and reaction buffer without inhibitor were used as positive and negative controls, respectively. Oseltamivir acid at 100 μg/mL inhibited neuraminidase activity by approximately 94%.

## Figures and Tables

**Table 1:** ^1^H NMR data for compounds **1**−**3** (500 MHz, CDCl_3_, *δ* in ppm).

Pos.	1	2	3
	*δ*_H_ Mult. (*J* in Hz)	*δ*_H_ Mult. (*J* in Hz)	*δ*_H_ Mult. (*J* in Hz)
2	9.60, s	8.70, s	8.30, s
4	6.95, d (7.8)	6.97, d (7.8)	6.94, d (8.0)
5	7.43, t (7.7)	7.49, t (7.7)	7.46, m
6	7.25, t (7.7)	7.32, t (7.7)	7.25, m
7	7.94, d (7.8)	7.99, d (7.8)	7.94, d (7.9, 1.5)
11	4.28, dd (8.5, 6.3)	4.32, dd (8.6, 6.3)	
12	3.44, dd (14.2, 8.5) 3.14, m	3.47, dd (14.4, 8.6) 3.18, m	6.65, s
14/18	7.12, d (8.3)	7.18, d (8.3)	7.36, d (8.8)
15/17	6.77, d (8.3)	6.82, d (8.3)	6.82, d (8.8)
20	9.26, s	8.38, s	7.96, s
22	7.06, d (8.0)	7.05, d (8.0)	7.00, d (8.0)
23	7.50, t (7.6)	7.56, t (7.7)	7.43, m
24	7.30, t (7.6)	7.32, t (7.7)	7.23, m
25	8.10, d (8.0)	8.14, d (8.0)	7.99, d (7.9, 1.6)
29	4.22, dd (11.0, 6.3)	4.25, dd (11.1, 6.1)	
30	2.79, dd (13.9, 6.3) 2.66, dd (13.9, 11.0)	2.82, dd (13.9, 6.1) 2.69, dd (13.9, 11.1)	6.90, s
32/36	6.92, d (8.6)	7.18, d (8.6)	7.32, d (8.8)
33/35	6.79, d (8.6)	6.85, d (8.6)	6.90, d (8.8)
37	3.14, s	3.18, s	3.48, s
38	2.93, s	2.97, s	3.22, s
39	3.73, s	3.79, s	3.77, s
40	3.73, s	3.79, s	3.83, s

**Table 2:** ^13^C NMR data for compounds **1**−**3** (125 MHz, CDCl_3_, *δ* in ppm).

Pos.	1 *δ*_C_, Type	2 *δ*_C_, Type	3 *δ*_C_, Type
1	172.0, C	171.6, C	172.0, C
3	126.9, C	127.0 C	127.1 C
4	120.8, CH	120.7, CH	120.8, CH
5	132.4, CH	132.5, CH	132.4, CH
6	125.3, CH	125.5, CH	125.5, CH
7	131.5, CH	131.6, CH	131.5, CH
8	135.7, C	135.6, C	135.9, C
9	168.5, C	168.5, C	167.0, C
11	56.7, CH	56.7, CH	130.6, C
12	31.5, CH_2_	31.5, CH_2_	133.1, CH
13	128.4, C	128.4, C	124.7, C
14/18	129.9, 2CH	130.2, 2CH	130.7, 2CH
15/17	114.4, 2CH	114.5, 2CH	114.6, 2CH
16	158.6, C	158.6, C	160.7, C
19	170.3, C	169.9, C	168.3, C
21	127.5, C	127.6, C	127.9, C
22	120.3, CH	120.1, CH	120.4, CH
23	132.8, CH	132.8, CH	132.8, CH
24	125.2, CH	125.3, CH	125.1, CH
25	131.8, CH	132.0, CH	131.6, CH
26	134.8, C	134.7 C	134.7, C
27	166.1, C	166.1, C	166.6, C
29	68.7, CH	68.8, CH	131.1, C
30	33.8, CH_2_	33.9, CH_2_	131,5, CH
31	127.6, C	127.6, C	125.8, C
32/36	130.2, 2CH	130.2, 2CH	131.6, 2CH
33/35	114.2, 2CH	114.2, 2CH	114.3, 2CH
34	158.8, C	158.9, C	161.0, C
37	29.4, CH_3_	29.4, CH_3_	36.1, CH_3_
38	39.8, CH_3_	39.8, CH_3_	37.9, CH_3_
39	55.3, CH_3_	55.3, CH_3_	55.5, CH_3_
40	55.3, CH_3_	55.3, CH_3_	55.5, CH_3_

**Table 3:** ^1^H data for compounds **4**−**7** (500 MHz, *δ* in ppm, *J* in Hz).

Pos.	4 ^a^ *δ*_H_ Mult.	5 ^b^ *δ*_H_ Mult.	6 ^b^ *δ*_H_ Mult.	7 ^b^ *δ*_H_ Mult.
2-NH	9.02, brs			8.74, s
3				4.00, dt (8.4, 6.1)
3-OH	6.31, d (3.8)			
5-NH		7.85, s	7.79, s	
7	7.73, dd (8.2, 1.1)	6.97, d (8.0)	6.91, d (8.0)	7.02, d (8.1)
8	7.87, ddd (8.4, 7.1, 1.6)	7.43, td (7.7, 1.6)	7.45, td (8.0, 1.6)	7.50, td (7.7, 1.6)
9	7.59, ddd (8.2, 7.1, 1.1)	7.25, overlapped	7.24, t (7.6)	7.26, overlapped
10	8.20, dd (8.0, 1.6)	7.94, dd (8.0, 1.6)	8.00, dd (8.0, 1.6)	7.92, dd (8.1, 1.6)
12		6.65, s	7.79, s	3.01, dd (14.6, 8.4) 3.35, dd (14.6, 6.1)
14/18	5.40, m	7.37, d (8.8)	7.33, d (8.8)	7.19, d (8.5)
15/17	3.58, dd (12.6, 3.8) 4.07, d (12.6)	6.83, d (8.8)	6.83, d (8.8)	6.83, d (8.5)
17	2.79, dd (14.5, 1.9) 2.48, dd (14.5, 4.3)			
18-NH	5.92, s			
19		3.49, d (3.2)	3.22, s	3.77, s
20	6.67, dd (8.1, 1.2)	3.79, s	3.83, s	
21	7.04, ddd (8.4, 7.1, 1.5)			
22	6.61, td (7.4, 1.2)			
23	7.25, dd (7.8, 1.5)			

^a^ Measured in DMSO-*d*_6_; ^b^ Measured in CDCl_3_.

**Table 4:** ^13^C data for compounds **4**−**7** (500 MHz, *δ* in ppm, *J* in Hz).

Position	4 ^a^	5 ^b^	6 ^b^	7 ^b^
	*δ*_C_ Type	*δ*_c_ Type	*δ*_C_ Type	*δ*_C_ Type
1	169.4, C	166.6, C	167.1, C	168.9, C
3	70.7, C	130.5, C	130.8, C	53.9, CH
4	153.8, C	168.1, C	172.0, C	171.1, C
6	147.0, C	127.1, C	125.8, C	125.7, C
7	127.4, CH	120.7, CH	120.4, CH	121.2, CH
8	134.8, CH	132.4, CH	132.8, CH	133.3, CH
9	127.1, CH	125.6, CH	125.2, CH	125.4, CH
10	126.4, CH	131.8, CH	131.6, CH	131.5, CH
11	120.3, C	134.6, C	135.8, C	135.8, C
12	158.4, C	133.2, CH	131.6, CH	33.7, CH_2_
13		124.7, C	124.7, C	128.1, C
14/18	52.3, CH	130.8, 2CH	131.5, 2CH	130.5, 2CH
15/17	37.6, CH_2_	114.3, 2CH	114.6, 2CH	114.0, 2CH
16	59.3, C	160.7, C	161.0, C	158.9, C
17	40.0, CH_2_			
19	143.1, C	37.9, CH_3_	36.1, CH_3_	55.4, CH_3_
20	114.2, CH	55.5, CH_3_	55.5, CH_3_	
21	128.4, CH			
22	116.1, CH			
23	129.1, CH			
24	123.7, C			

^a^ Measured in DMSO-*d*_6_; ^b^ Measured in CDCl_3_.

**Table 5:** Neuraminidase inhibitory activity of compounds **1**–**10** at a concentration of 100 µg/mL.

Compounds	Inhibition Rate/% Mean ± SD
**1**	52.90 ± 0.37
**2**	43.16 ± 1.23
**3**	58.67 ± 3.51
**4**	74.01 ± 1.32
**5**	85.40 ± 0.85
**6**	63.65 ± 0.67
**7**	57.78 ± 0.44
**8**	43.85 ± 1.41
**9**	51.67 ± 0.42
**10**	67.54 ± 0.87

## Data Availability

The original contributions presented in this study are included in the article/supplementary material. Further inquiries can be directed to the corresponding authors.

## Supplementary Materials

The following supporting information can be downloaded at: https://www.mdpi.com/article/10.3390/md23100377/s1, ITS sequence data of ITS sequence data of *Penicillium* sp. SCSIO41035, 1D and 2D NMR spectra, and HRESIMS data for the new compounds.
