# Supplementary material for: Novel Cyclic Tetrapeptides as Neuraminidase Inhibitors from a Sponge-Associated Penicillium sp. SCSIO41035
Source: Mar Drugs. 2025 Sep 26;23(10):377. doi: 10.3390/md23100377 (PMC12565300; doi:10.3390/md23100377)
Supplement: Supplementary file 1 [file marinedrugs-23-00377-s001.zip › marinedrugs-3853428-supplementary.pdf]

Supplementary Materials

Novel cyclic tetrapeptides as neuraminidase inhibitors from a sponge-associated *Penicillium* sp. SCSIO41035

Weihao Chen [1,2,†], Xiangliu Chen [1,2,†], Mengjing Cong [1,2], Jianglian She [1,2], Xiaoyan Pang [1,2,3], Shengrong Liao [1,2,3], Bin Yang [1,2,3], Xuefeng Zhou [1,2,3], Yonghong Liu [1,2,3,*], Fuquan Xu [4,*] and Junfeng Wang [1,2,3,*]

[1] State Key Laboratory of Tropical Oceanography, Guangdong Key Laboratory of Marine Materia Medica, South China Sea Institute of Oceanology, Chinese Academy of Sciences, Guangzhou 510301, China; chenweihao17@mails.ucas.ac.cn (W.C.); chenxiangliu23@mails.ucas.ac.cn (X.C.); c3021632921@163.com (M.C.); shejianglian20@mails.ucas.ac.cn (J.S.); xypang@scsio.ac.cn (X.P.); ljrss@126.com (S.L.); bingo525@163.com (B.Y.); xfzhou@scsio.ac.cn (X.Z.)

[2] University of Chinese Academy of Sciences, 19 Yuquan Road, Beijing 100049, China

[3] Sanya Institute of Ocean Eco-Environmental Engineering, Sanya 572000, China

[4] Jiangsu Key Laboratory of Marine Biotechnology, Jiangsu Ocean University, Lianyungang 222000, China

* Correspondence: yonghongliu@scsio.ac.cn (Y.L.); sdfuquanxu@126.com (F.X.); wangjunfeng@scsio.ac.cn (J.W.)

† These authors contributed equally to this work.

List of supporting information

The ITS gene sequences data for *Penicillium* sp. SCSIO41035 4

Figure S1. The ^{1}H NMR spectrum of **1** in $CDCl_3$. 4

Figure S2. The ^{13}C NMR spectrum of **1** in $CDCl_3$. 5

Figure S3. The HSQC spectrum of **1** in $CDCl_3$. 5

Figure S4. The ^{1}H-^{1}H COSY spectrum of **1** in $CDCl_3$. 6

Figure S5. The HMBC spectrum of **1** in $CDCl_3$. 6

Figure S6. The UV (left) and ECD (right) spectra of **1** in MeOH. 7

Figure S7. The HRESIMS spectrum of **1**. 7

Figure S8. The ^{1}H NMR spectrum of **2** in $CDCl_3$. 8

Figure S10. The HSQC spectrum of **2** in $CDCl_3$. 9

Figure S11. The ^{1}H-^{1}H COSY spectrum of **2** in $CDCl_3$. 9

Figure S12. The HMBC spectrum of **2** in $CDCl_3$. 10

Figure S13. The HRESIMS spectrum of **2**. 10

Figure S14. The UV (left) and ECD (right) spectra of **2** in MeOH. 11

Figure S15. The ^{1}H NMR spectrum of **3** in $CDCl_3$. 11

Figure S16. The ^{13}C NMR spectrum of **3** in $CDCl_3$. 12

Figure S17. The NOESY spectrum of **3** in $CDCl_3$. 12

Figure S18. The HSQC spectrum of **3** in $CDCl_3$. 13

Figure S19. The ^{1}H-^{1}H COSY spectrum of **3** in $CDCl_3$. 13

Figure S20. The HMBC spectrum of **3** in $CDCl_3$. 14

Figure S21. The UV spectrum of **3** in MeOH. 14

Figure S22. The HRESIMS spectrum of **3**. 15

Figure S23. The ^{1}H NMR spectrum of **4** in DMSO-d_6. 15

Figure S24. The ^{13}C NMR spectrum of **4** in DMSO-d_6. 16

Figure S25. The HSQC spectrum of **4** in DMSO-d_6. 16

Figure S26. The ^{1}H-^{1}H COSY spectrum of **4** in DMSO-d_6. 17

Figure S27. The HMBC spectrum of **4** in DMSO-d_6. 17

Figure S28. The NOESY spectrum of **4** in DMSO-d_6. 18

Figure S29. The UV (left) and ECD (right) spectra of **4** in MeOH. 18

Figure S30. The HRESIMS spectrum of **4**. 19

Figure S31. The ^{1}H NMR spectrum of **5** in $CDCl_3$. 19
Figure S32. The ^{13}C NMR spectrum of **5** in $CDCl_3$. 20
Figure S33. The HSQC spectrum of **5** in $CDCl_3$. 20
Figure S34. The NOESY spectrum of **5** in $CDCl_3$. 21
Figure S35. The HMBC spectrum of **5** in $CDCl_3$. 21
Figure S36. The UV spectrum of **5** in MeOH. 22
Figure S37. The HRESIMS spectrum of **5**. 22
Figure S38. The ^{1}H NMR spectrum of **6** in $CDCl_3$. 23
Figure S39. The ^{13}C NMR spectrum of **6** in $CDCl_3$. 23
Figure S40. The HSQC spectrum of **6** in $CDCl_3$. 24
Figure S41. The NOESY spectrum of **6** in $CDCl_3$. 24
Figure S42. The HMBC spectrum of **6** in $CDCl_3$. 25
Figure S43. The UV spectrum of **6** in MeOH. 25
Figure S44. The HRESIMS spectrum of **6**. 26
Figure S45. The ^{1}H NMR spectrum of **7** in $CDCl_3$. 26
Figure S46. The ^{13}C NMR spectrum of **7** in $CDCl_3$. 27
Figure S47. The HSQC spectrum of **7** in $CDCl_3$. 27
Figure S48. The ^{1}H-^{1}H COSY spectrum of **7** in $CDCl_3$. 28
Figure S49. The HMBC spectrum of **7** in $CDCl_3$. 28
Figure S50. The UV (left) and ECD (right) spectra of **7** in MeOH. 29
Figure S51. The HRESIMS spectrum of **7**. 29
Table S1. Energies of all calculated conformers at B3LYP/6-31G* level in vacuum or chloroform. 29

The ITS gene sequences data for *Penicillium* sp. SCSIO41035

ATCGGAGGTGAGGCTCTGGGTCCACCTCCCACCCGTGTTTATTTTACCTTGT
TGCTTCGGCGGGCCCGCCTTTACTGGCCGCCGGGGGGCTCACGCCCCCGG
GCCCGCGCCCGCCGAAGACCCCCTCGAACTCTGTCTGAAGATTGAAGTCT
GAGTGAAAATATAAATTATTTAAAACTTTCAACAACGGATCTCTTGGTTCCG
GCATCGATGAAGAACGCAGCGAAATGCGATACGTAATGTGAATTGCAAATT
CAGTGAATCATCGAGTCTTTGAACGCACATTGCGCCCCCTGGTATTCCGGG
GGGCATGCCTGTCCGAGCGTCATTGCTGCCCTCAAGCCCGGCTTGTGTGTT
GGGCCCCGTCCTCCGATTCCGGGGGACGGGCCCGAAAGGCAGCGGCGGCA
CCGCGTCCGGTCCTCGAGCGTATGGGGCTTTGTCACCCGCTCTGTAGGCCC
GGCCGGCGCTTGCCGATCAACCCAAATTTTTATCCAGGTTGACCTCGGATC
AGGTAGGGGATACCCGCTGAACTTAAGCAATAAAAAAAGGGGGGGAAAA
AAAAAAATAATTATTTTT. (Genbank accession no. OM977045)

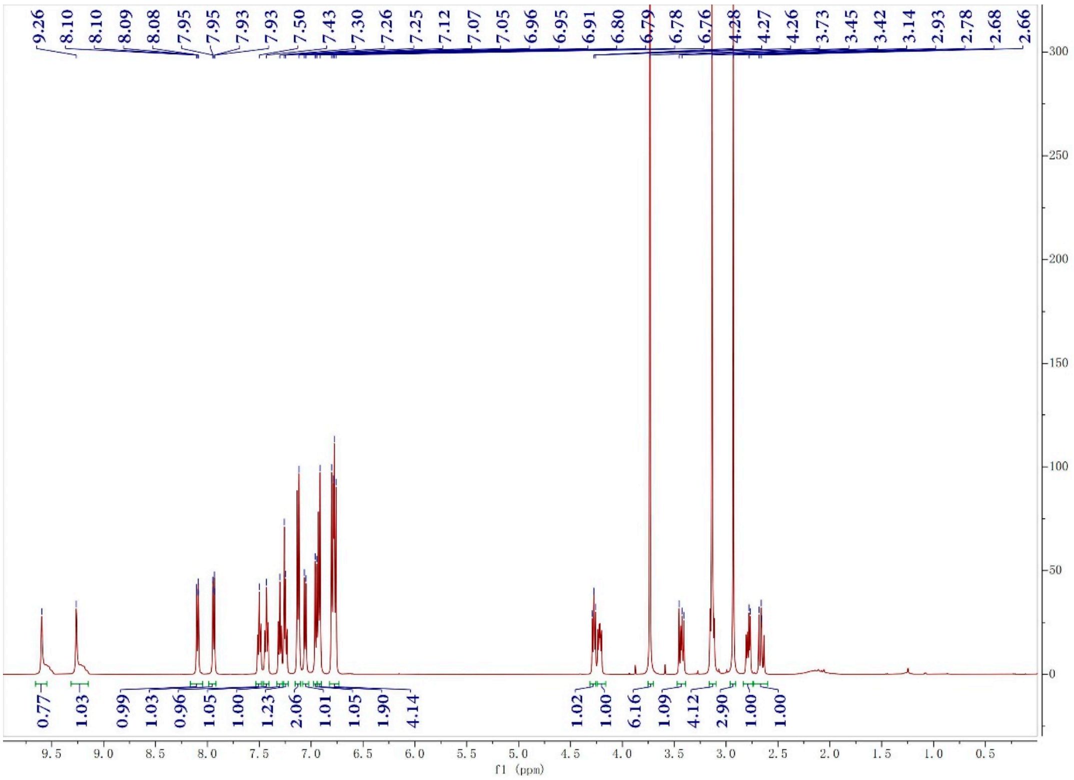

Figure S1. The ^{1}H NMR spectrum of **1** in $CDCl_3$.

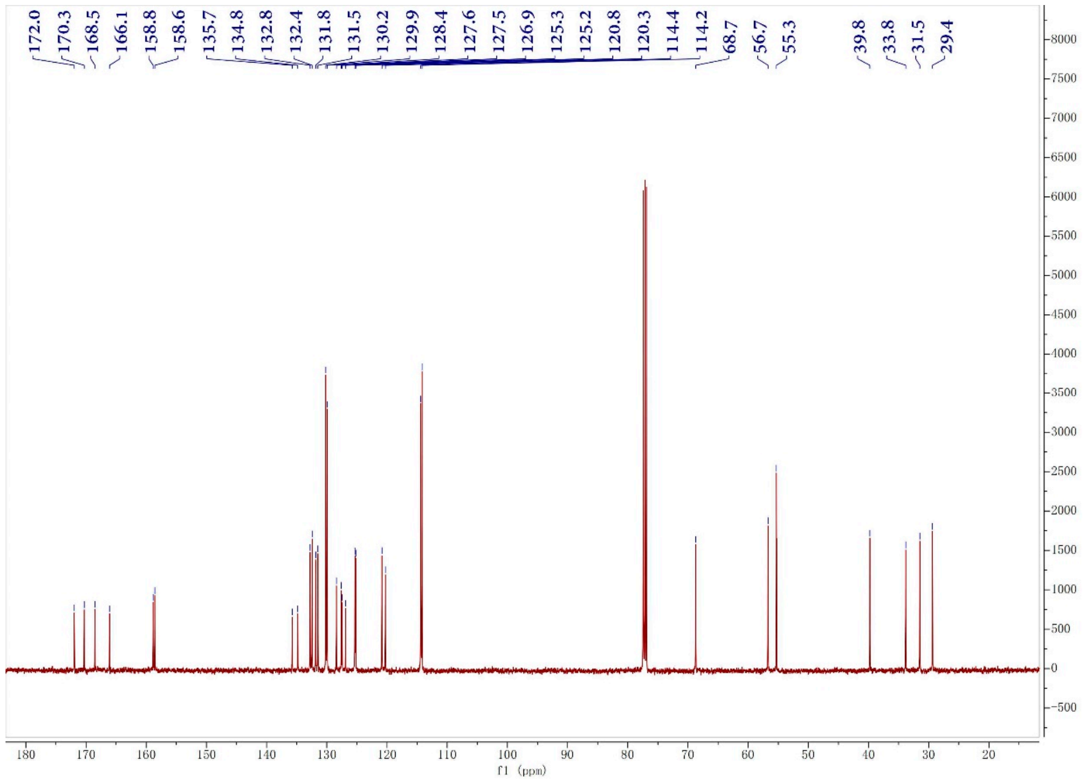

Figure S2. The ^{13}C NMR spectrum of **1** in $CDCl_3$.

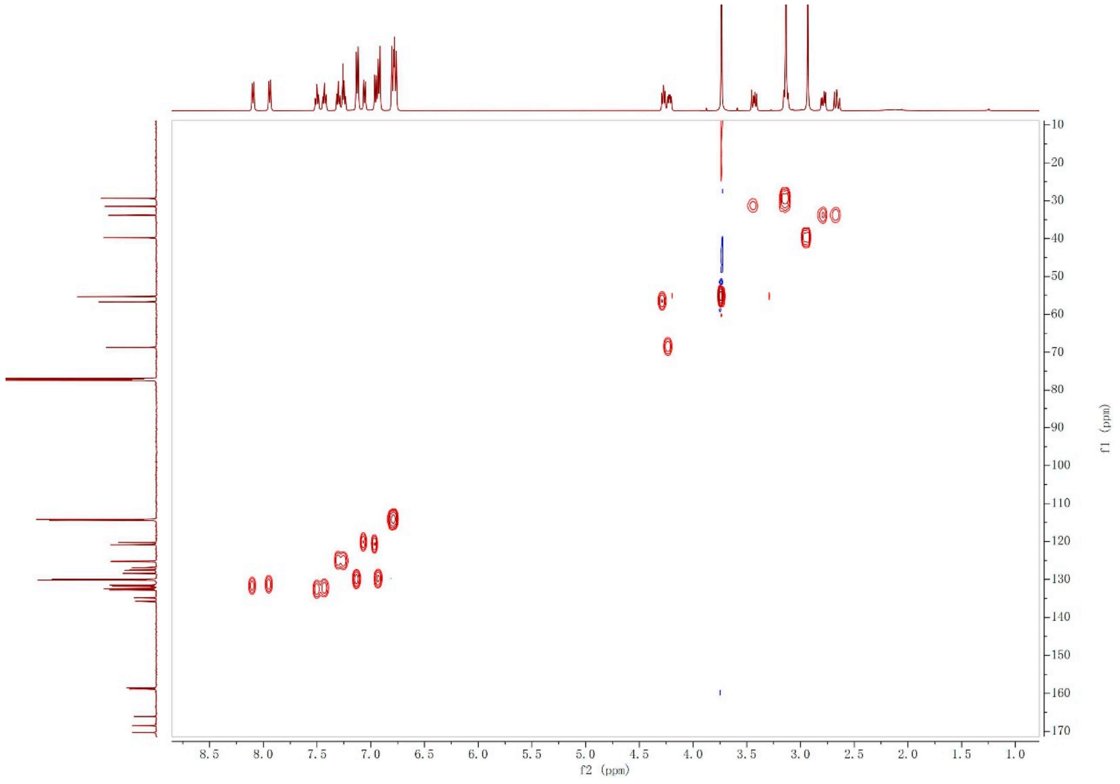

Figure S3. The HSQC spectrum of **1** in $CDCl_3$.

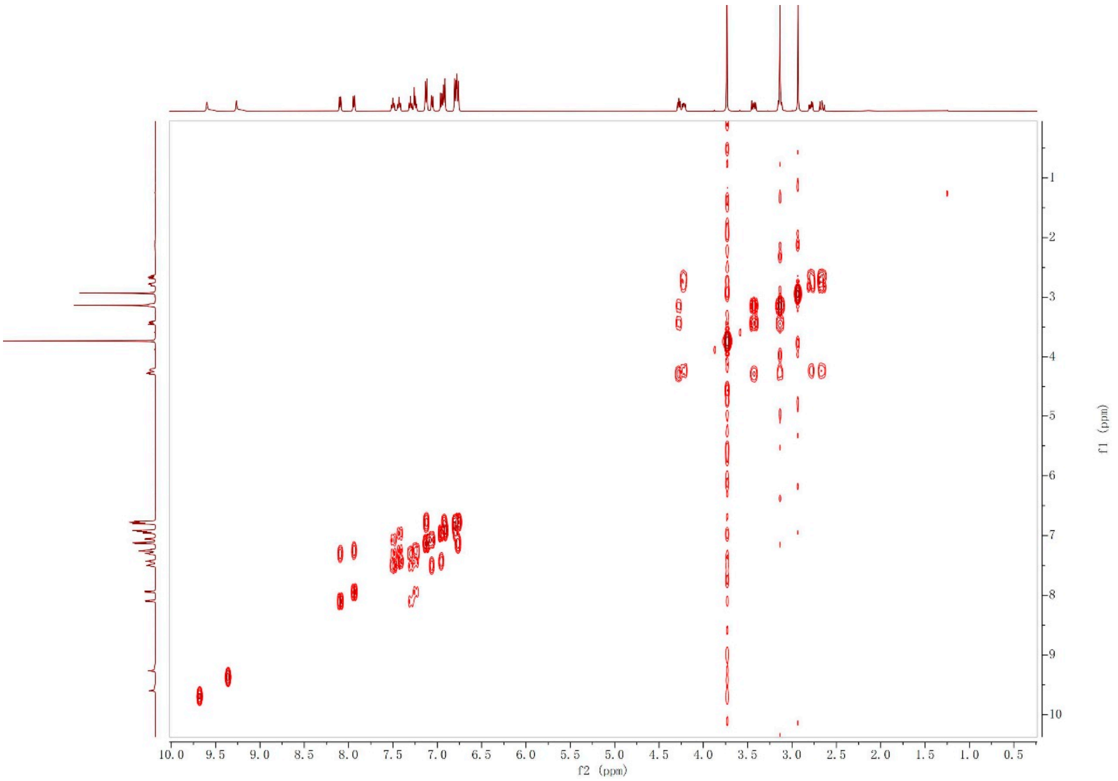

Figure S4. The ^{1}H-^{1}H COSY spectrum of **1** in $CDCl_3$.

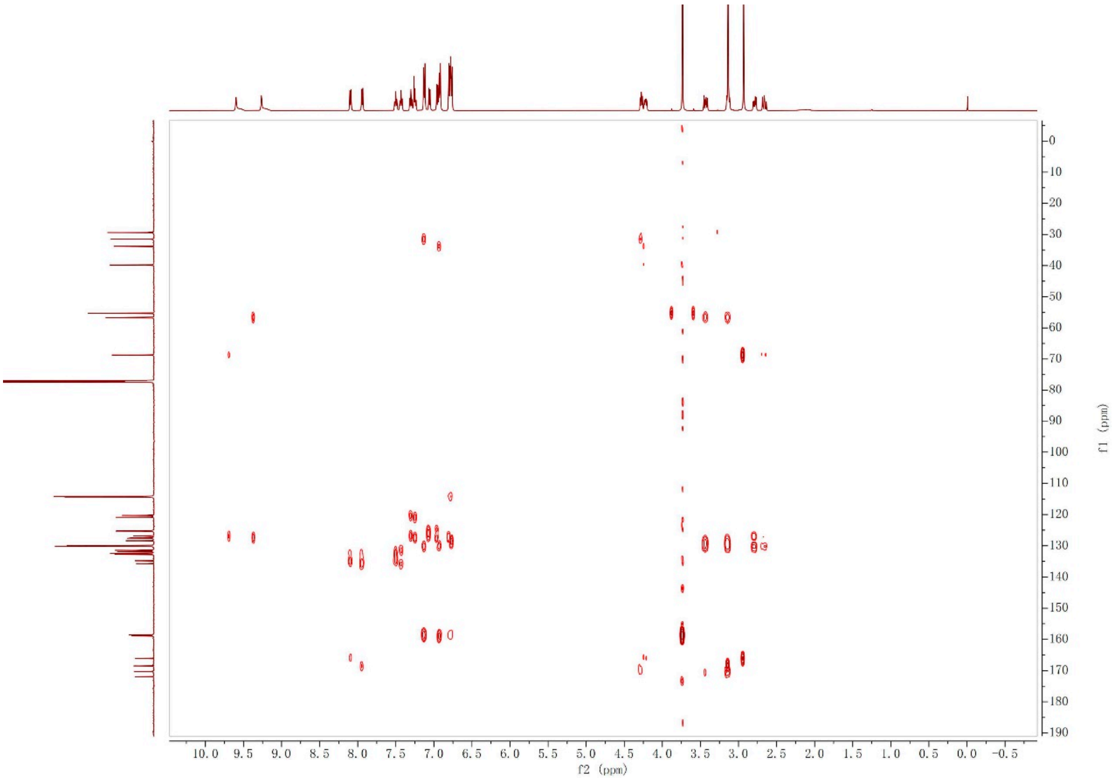

Figure S5. The HMBC spectrum of **1** in $CDCl_3$.

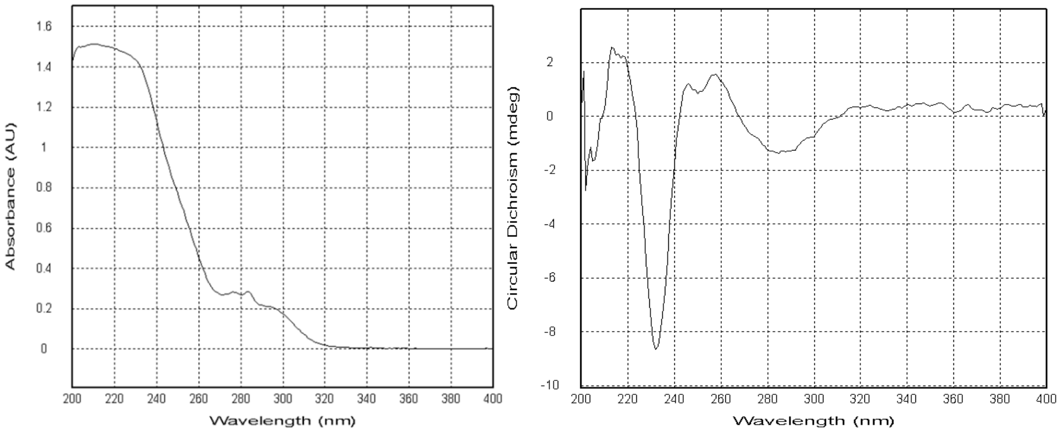

Figure S6. The UV (left) and ECD (right) spectra of **1** in MeOH.

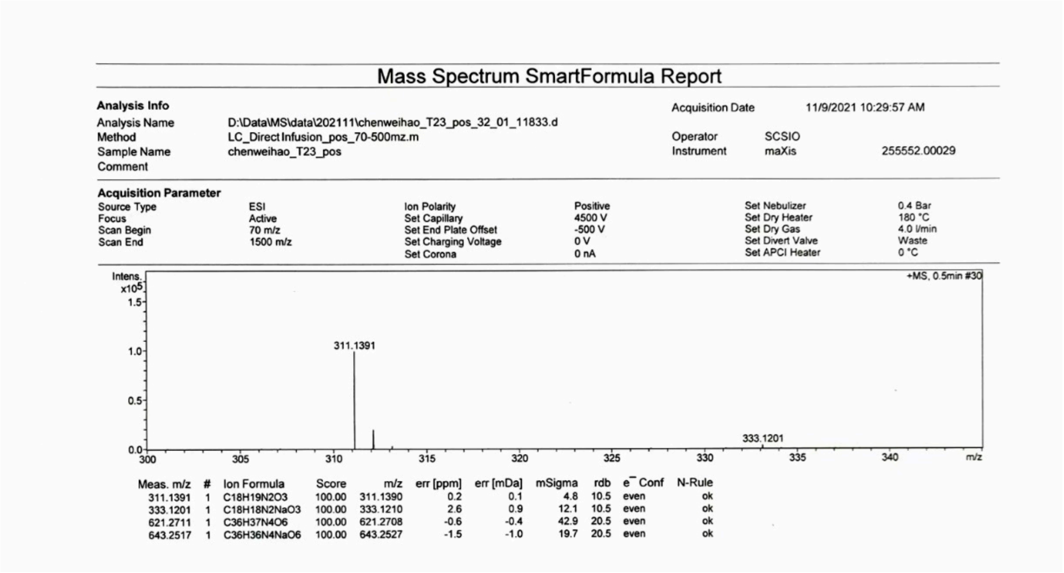

Mass Spectrum SmartFormula Report

Analysis Info

Analysis Name D:\Data\MS\data\202111\chenweihao_T23_pos_32_01_11833.d
Method LC_Direct Infusion_pos_70-500mz.m
Sample Name chenweihao_T23_pos
Comment

Acquisition Date 11/9/2021 10:29:57 AM
Operator SCSIO
Instrument maXis 255552.00029

Acquisition Parameter

Source Type	ESI	Ion Polarity	Positive	Set Nebulizer	0.4 Bar
Focus	Active	Set Capillary	4500 V	Set Dry Heater	180 °C
Scan Begin	70 m/z	Set End Plate Offset	-500 V	Set Dry Gas	4.0 l/min
Scan End	1500 m/z	Set Charging Voltage	0 V	Set Divert Valve	Waste
		Set Corona	0 nA	Set APCI Heater	0 °C

Meas. m/z	#	Ion Formula	Score	m/z	err [ppm]	err [mDa]	mSigma	rdb	e¯ Conf	N-Rule
311.1391	1	C18H19N2O3	100.00	311.1390	0.2	0.1	4.8	10.5	even	ok
333.1201	1	C18H18N2NaO3	100.00	333.1210	2.6	0.9	12.1	10.5	even	ok
621.2711	1	C36H37N4O6	100.00	621.2708	-0.6	-0.4	42.9	20.5	even	ok
643.2517	1	C36H36N4NaO6	100.00	643.2527	-1.5	-1.0	19.7	20.5	even	ok

Figure S7. The HRESIMS spectrum of **1**.

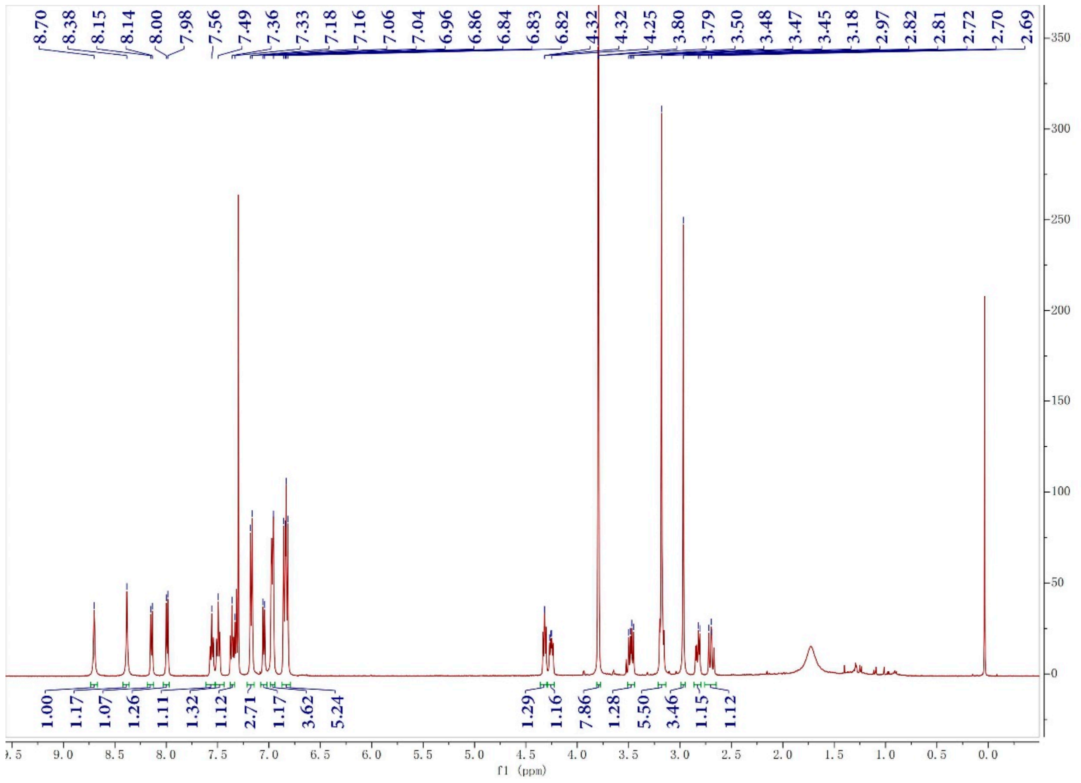

Figure S8. The ^{1}H NMR spectrum of **2** in $CDCl_3$.

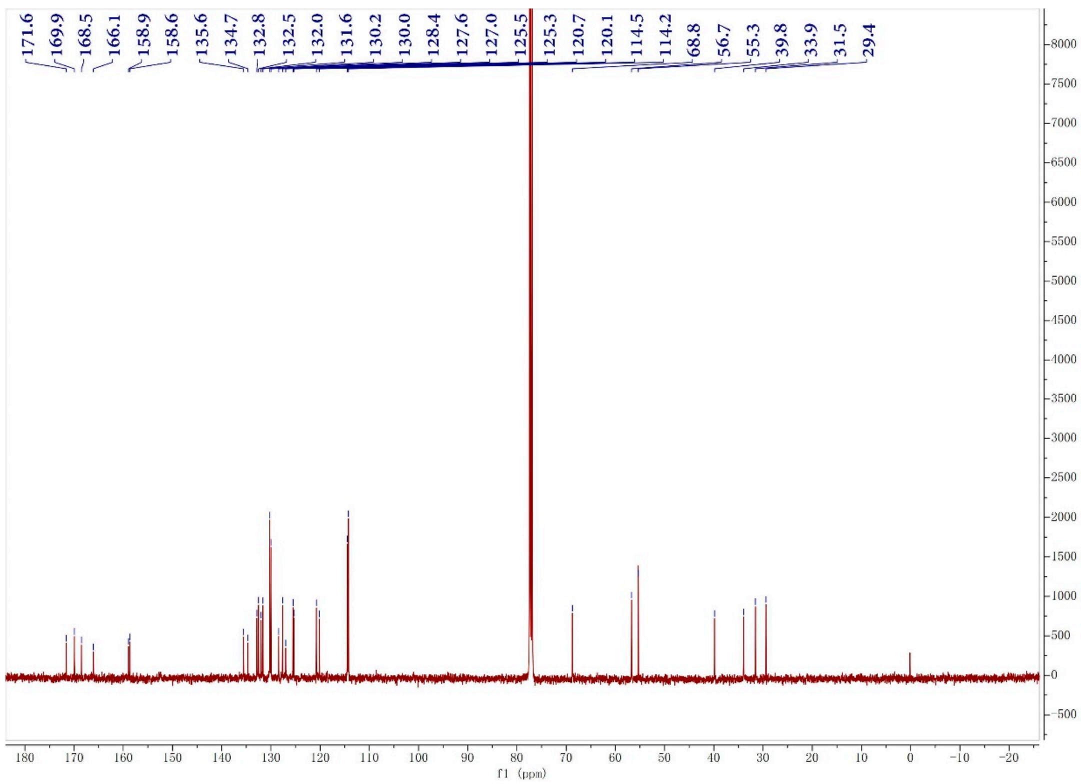

Figure S9. The ^{13}C NMR spectrum of **2** in $CDCl_3$.

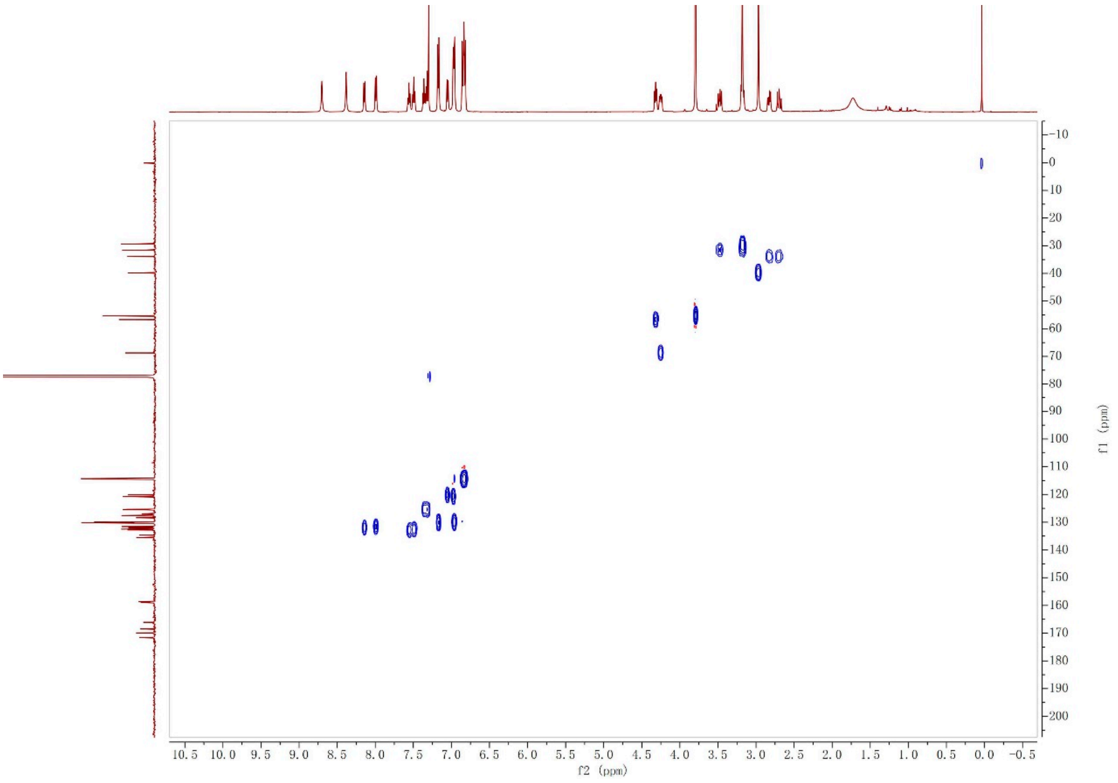

Figure S10. The HSQC spectrum of **2** in CDCl3.

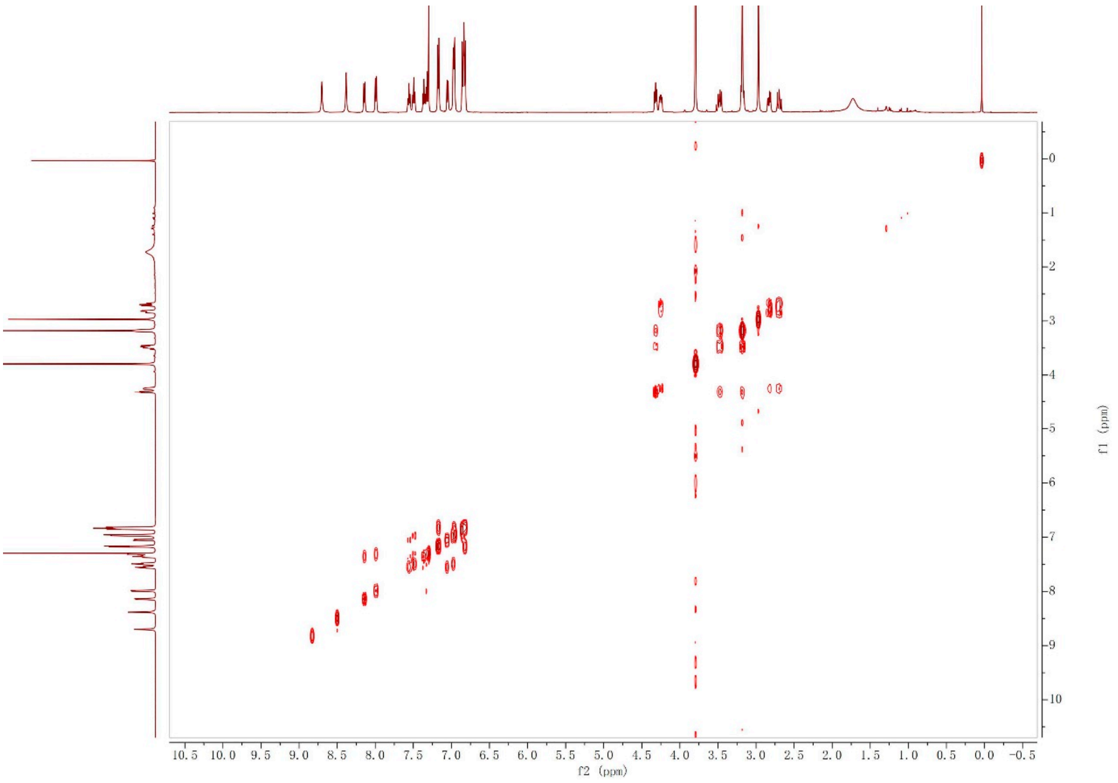

Figure S11. The ^{1}H-^{1}H COSY spectrum of **2** in CDCl3.

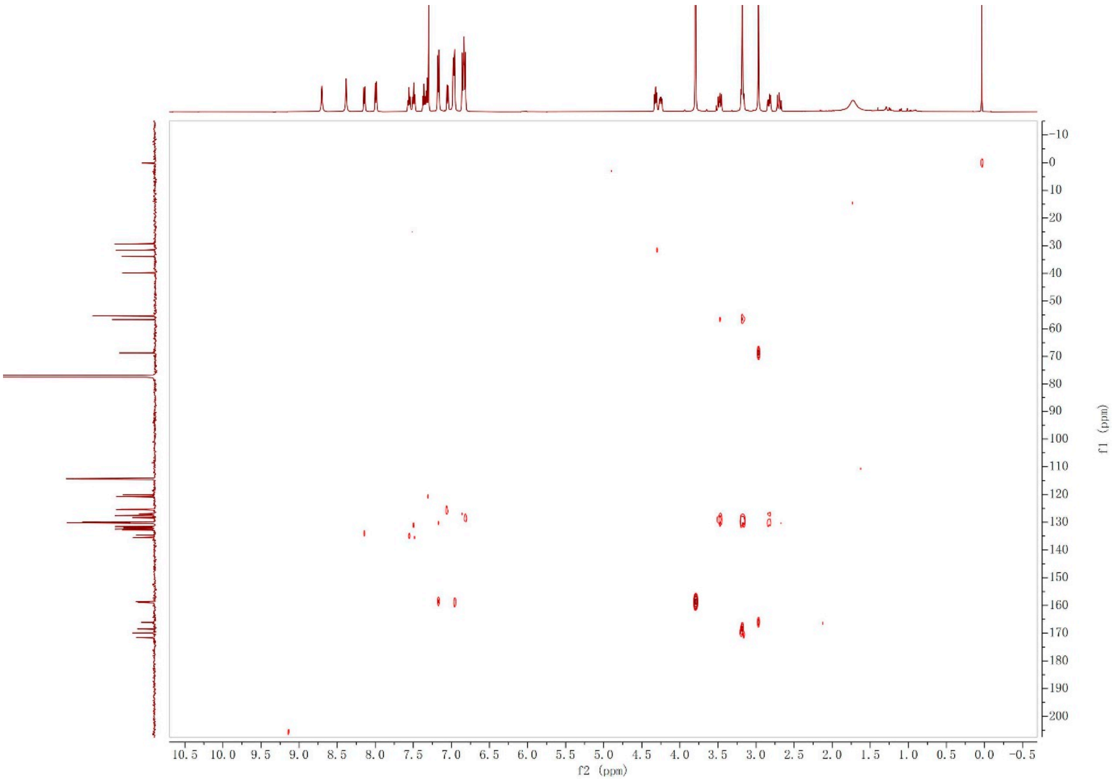

Figure S12. The HMBC spectrum of **2** in $CDCl_3$.

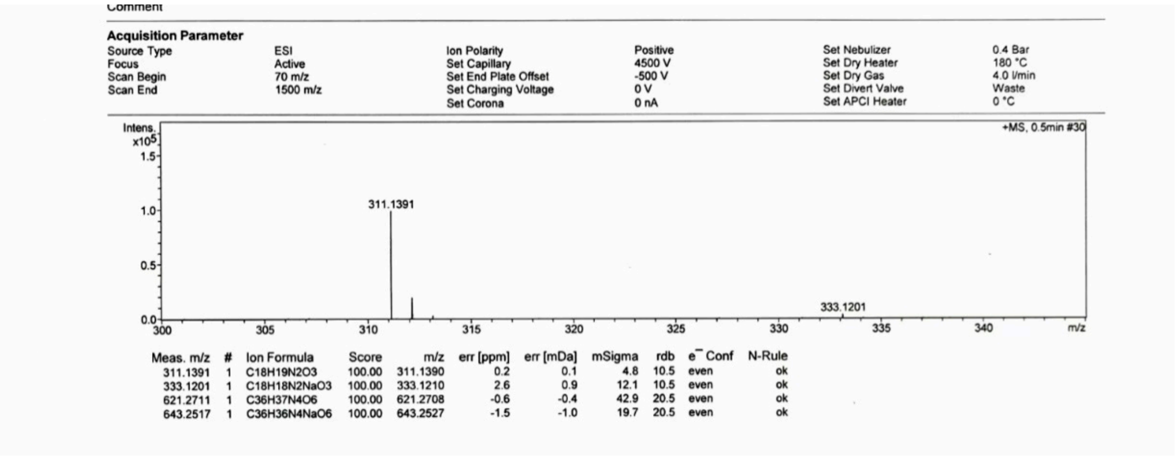

Figure S13. The HRESIMS spectrum of **2**.

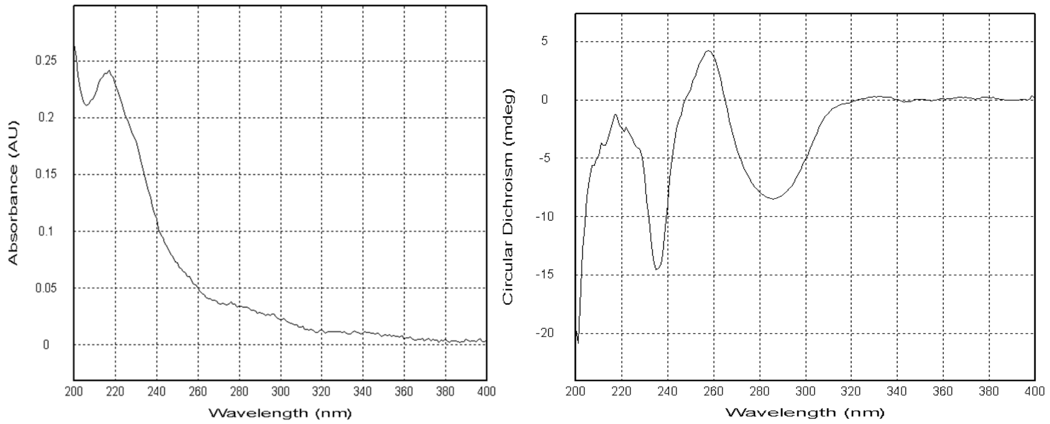

Figure S14. The UV (left) and ECD (right) spectra of **2** in MeOH.

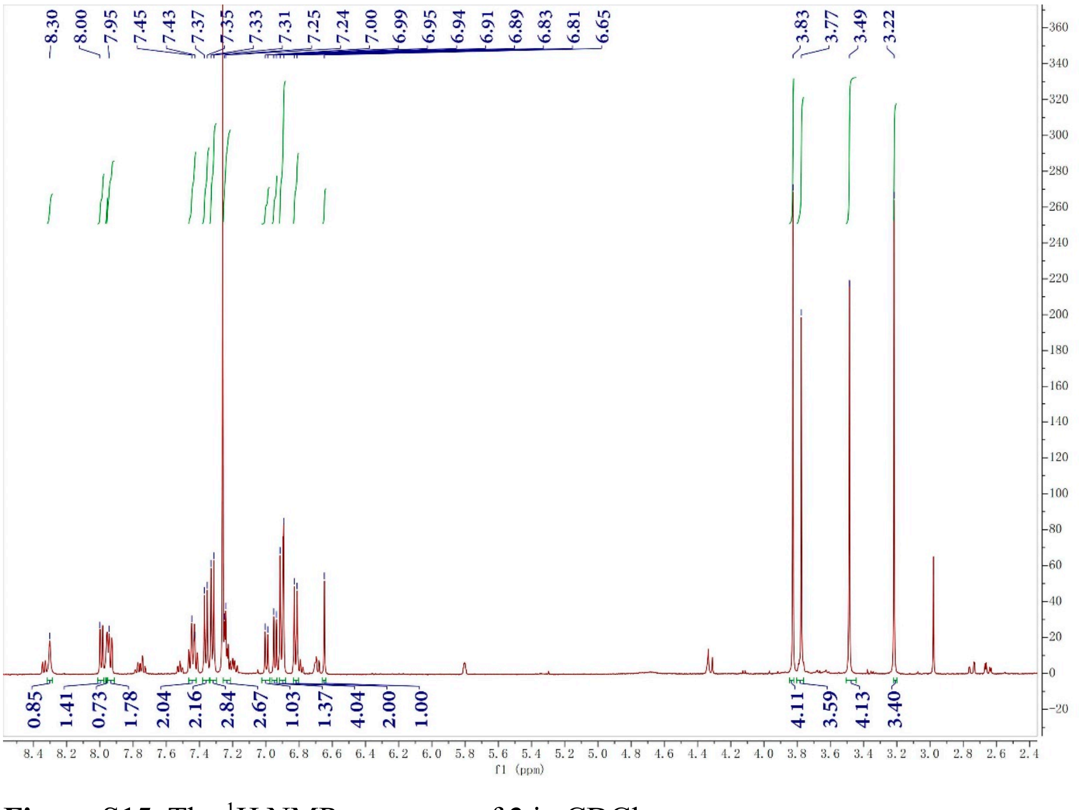

Figure S15. The ^{1}H NMR spectrum of **3** in $CDCl_3$.

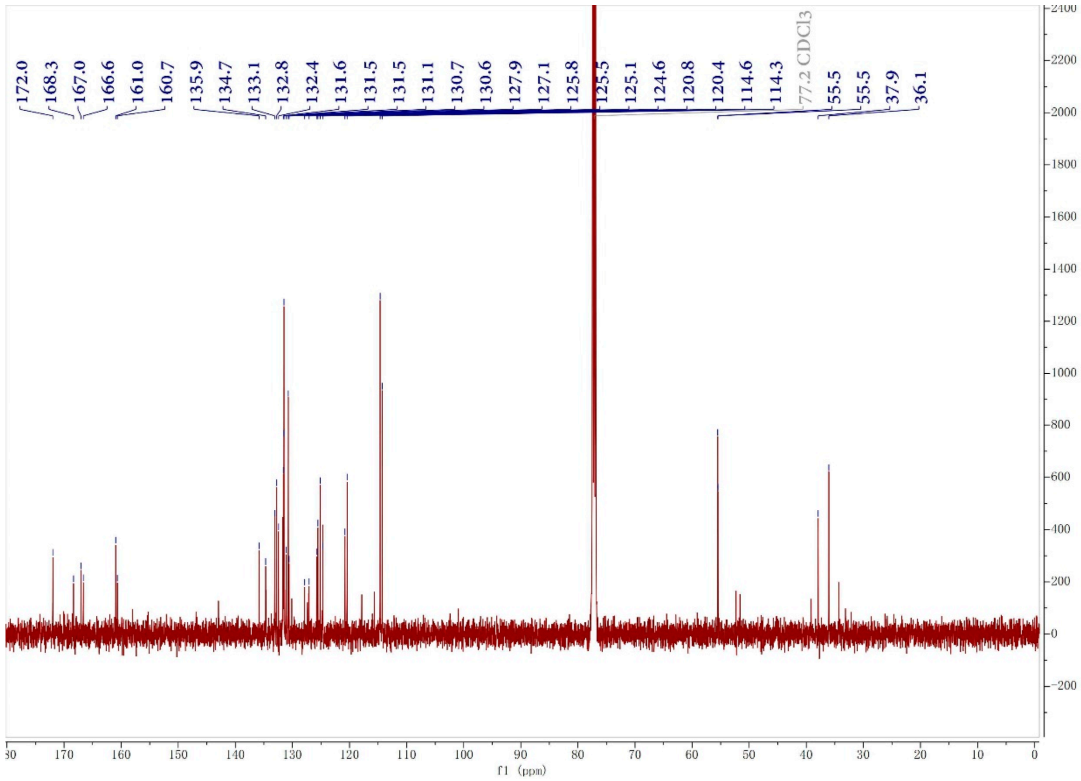

Figure S16. The ^{13}C NMR spectrum of **3** in $CDCl_3$.

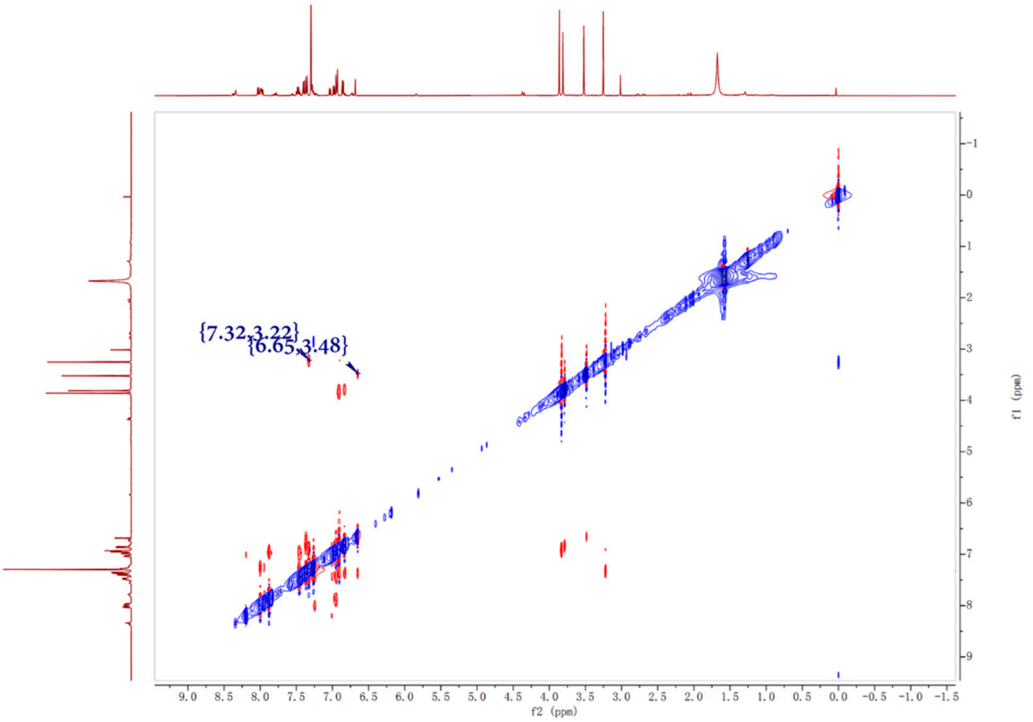

Figure S17. The NOESY spectrum of **3** in $CDCl_3$.

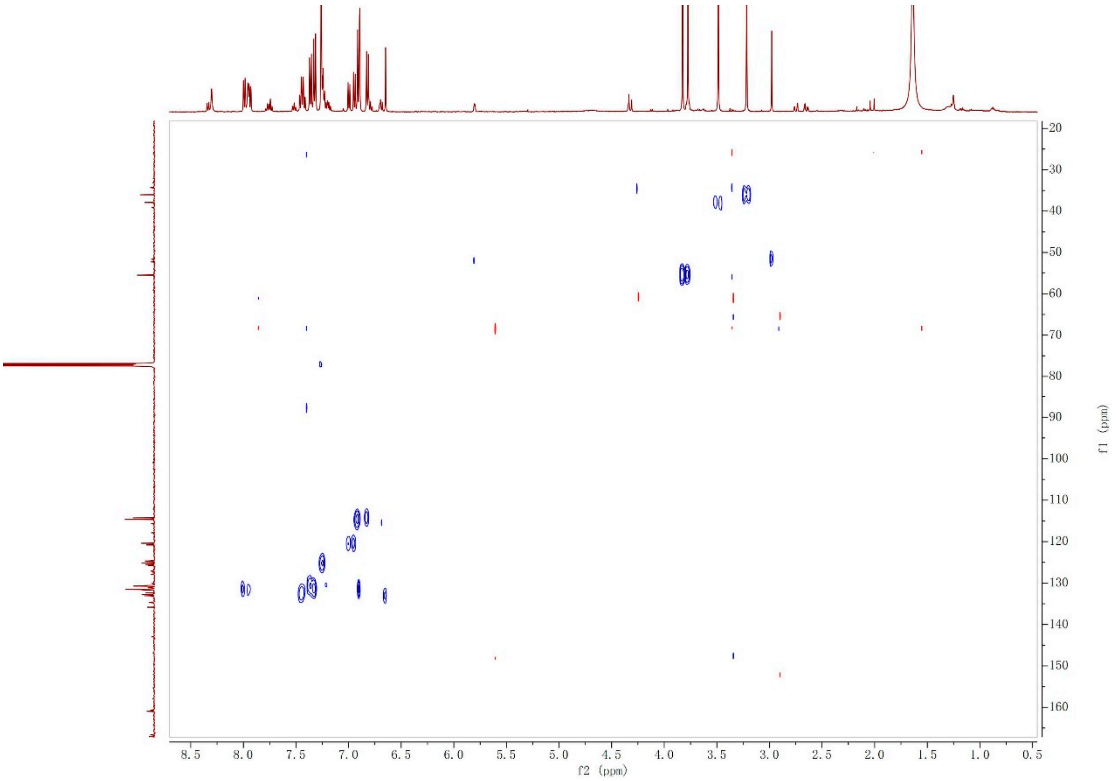

Figure S18. The HSQC spectrum of **3** in $CDCl_3$.

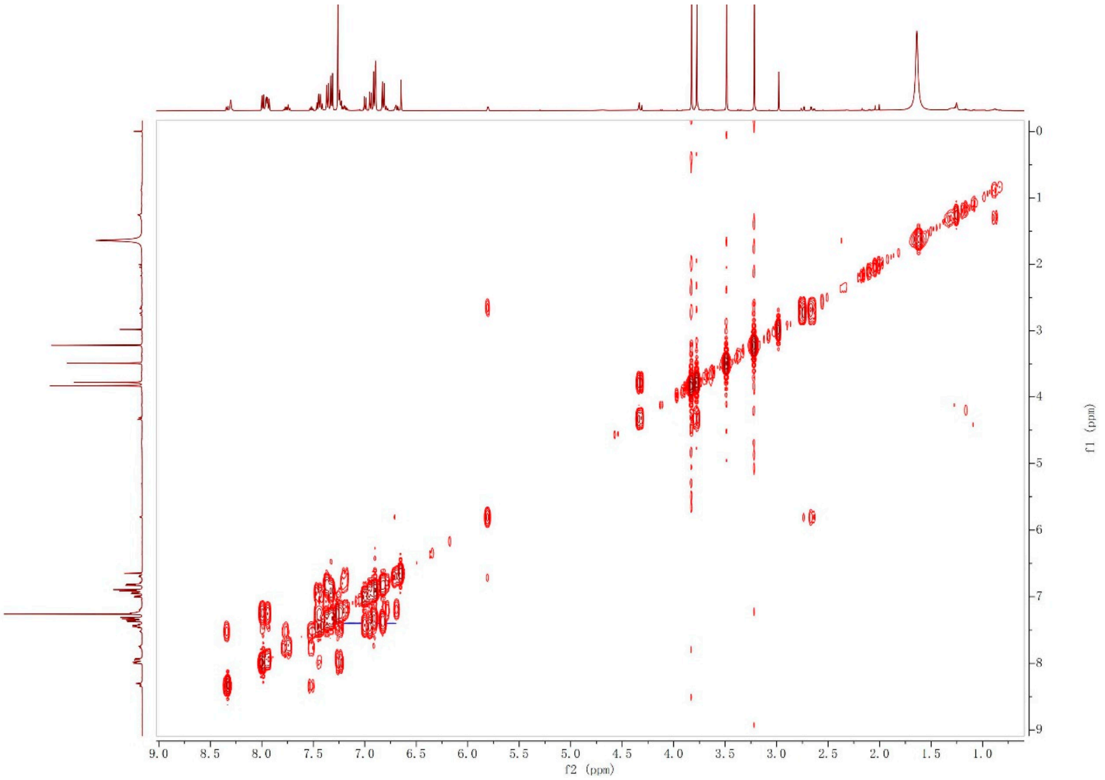

Figure S19. The ^1H-^1H COSY spectrum of **3** in $CDCl_3$.

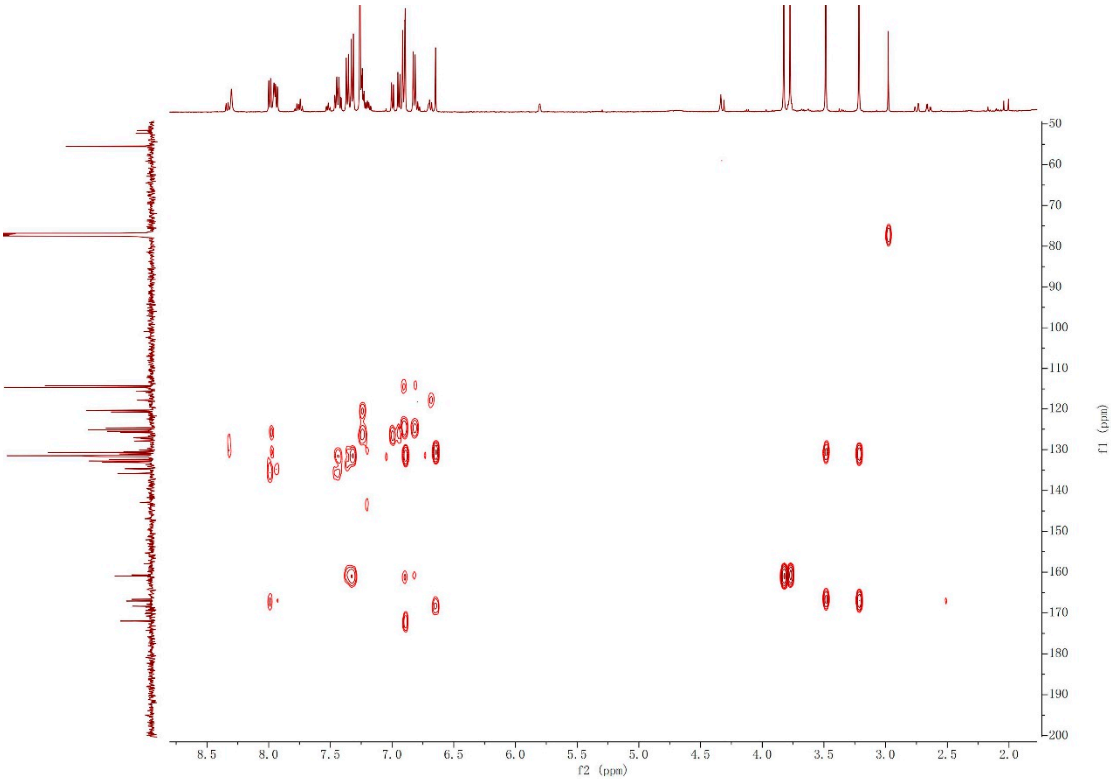

Figure S20. The HMBC spectrum of **3** in $CDCl_3$.

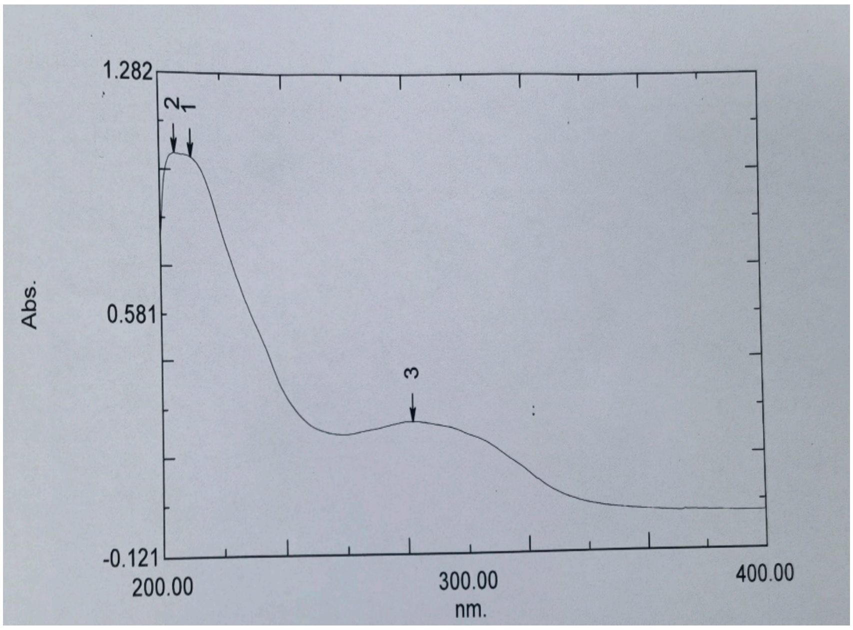

Figure S21. The UV spectrum of **3** in MeOH.

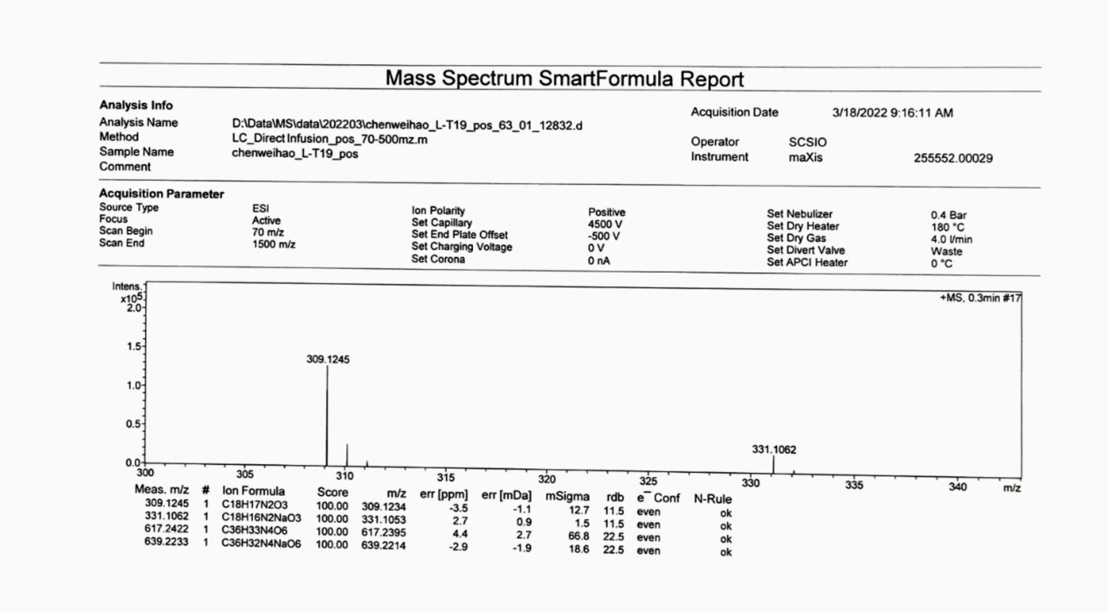

Mass Spectrum SmartFormula Report

Analysis Info

Analysis Name D:\Data\MS\data\202203\chenweihao_L-T19_pos_63_01_12832.d
Method LC_Direct Infusion_pos_70-500mz.m
Sample Name chenweihao_L-T19_pos
Comment

Acquisition Date 3/18/2022 9:16:11 AM
Operator SCSIO
Instrument maXis 255552.00029

Acquisition Parameter

Source Type ESI; Focus Active; Scan Begin 70 m/z; Scan End 1500 m/z
Ion Polarity Positive; Set Capillary 4500 V; Set End Plate Offset -500 V; Set Charging Voltage 0 V; Set Corona 0 nA
Set Nebulizer 0.4 Bar; Set Dry Heater 180 °C; Set Dry Gas 4.0 l/min; Set Divert Valve Waste; Set APCI Heater 0 °C

Meas. m/z	#	Ion Formula	Score	m/z	err [ppm]	err [mDa]	mSigma	rdb	e⁻ Conf	N-Rule
309.1245	1	C18H17N2O3	100.00	309.1234	-3.5	-1.1	12.7	11.5	even	ok
331.1062	1	C18H16N2NaO3	100.00	331.1053	2.7	0.9	1.5	11.5	even	ok
617.2422	1	C36H33N4O6	100.00	617.2395	4.4	2.7	66.8	22.5	even	ok
639.2233	1	C36H32N4NaO6	100.00	639.2214	-2.9	-1.9	18.6	22.5	even	ok

Figure S22. The HRESIMS spectrum of **3**.

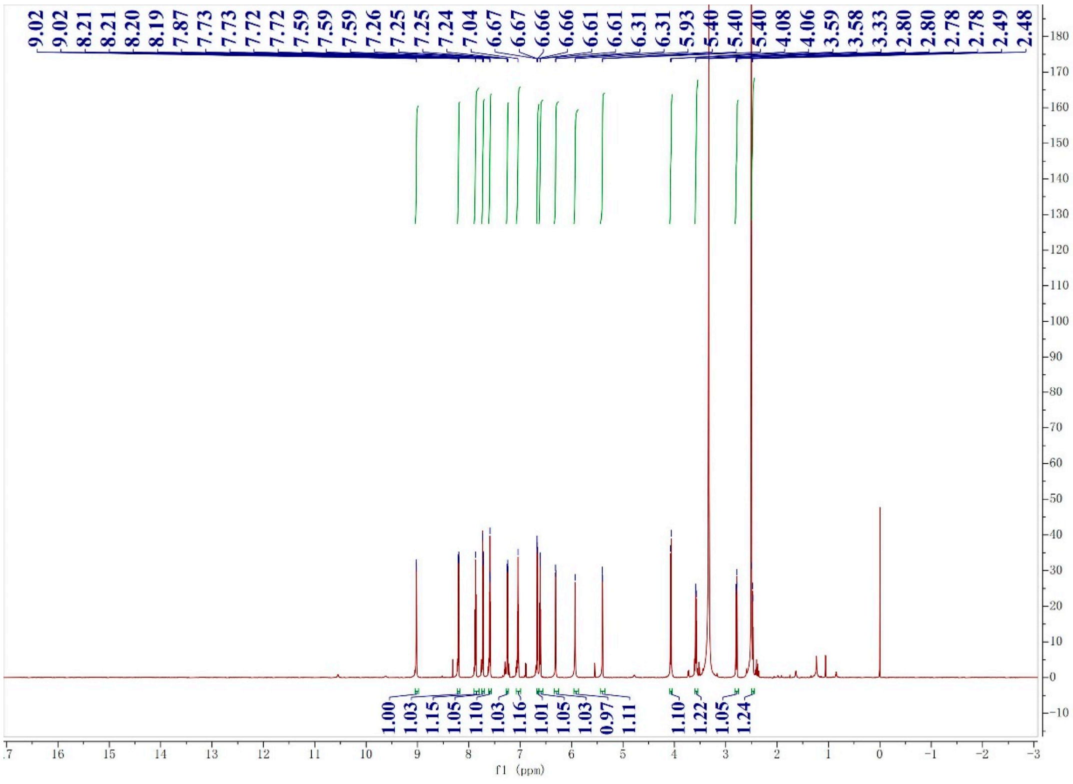

Figure S23. The ^{1}H NMR spectrum of **4** in DMSO-d_6.

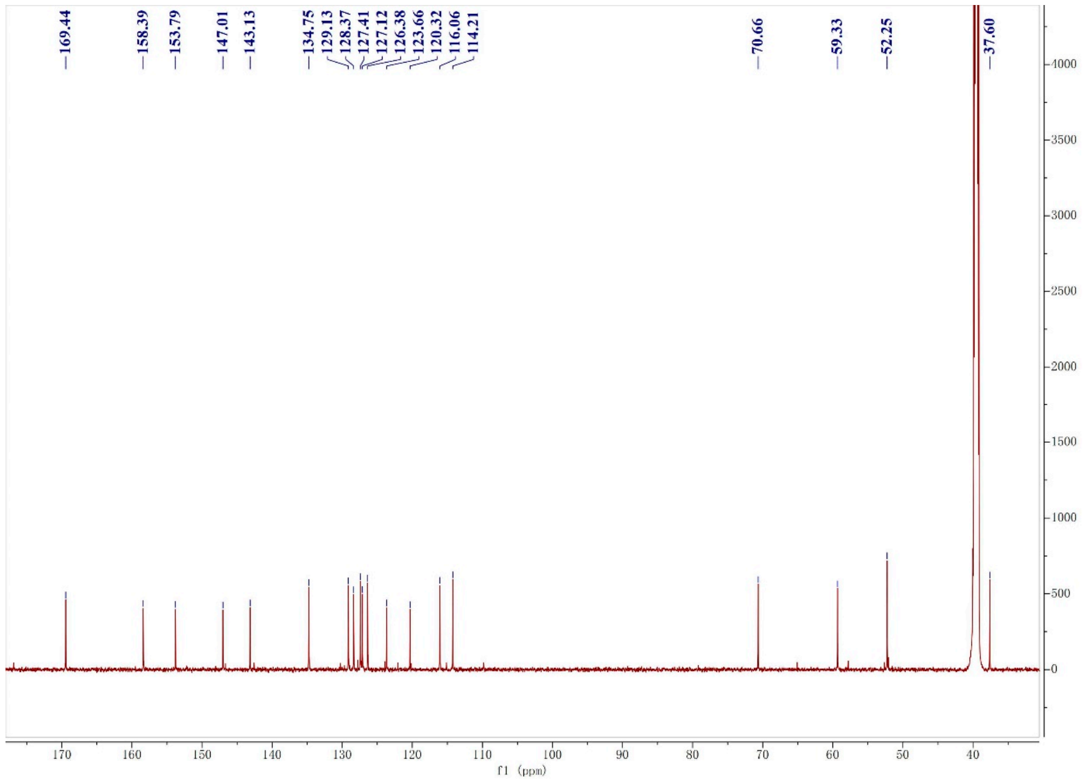

Figure S24. The ^{13}C NMR spectrum of **4** in DMSO-d_6.

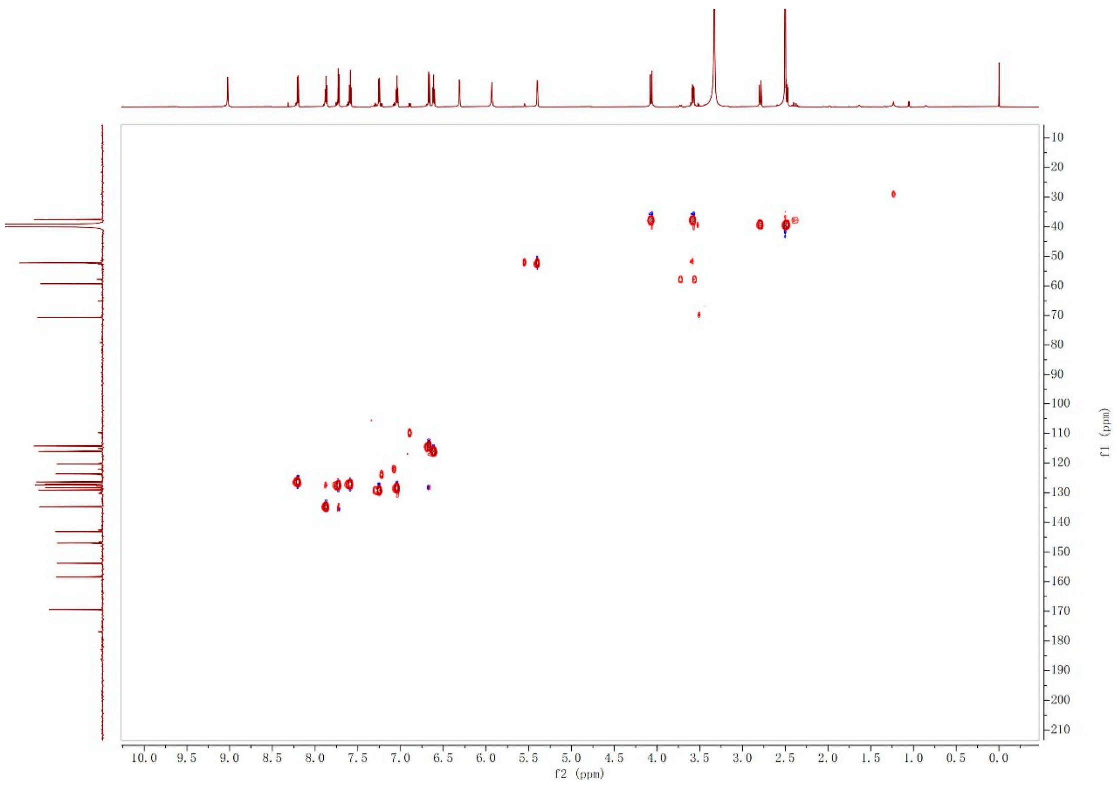

Figure S25. The HSQC spectrum of **4** in DMSO-d_6.

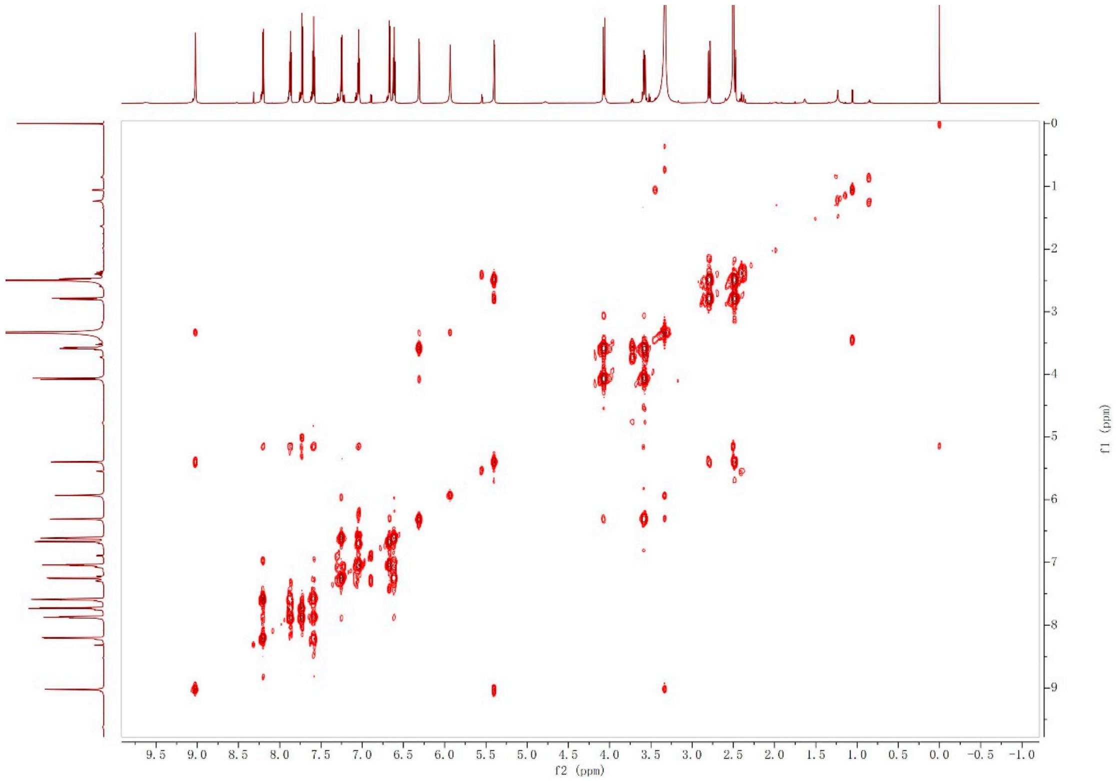

Figure S26. The ^{1}H-^{1}H COSY spectrum of **4** in DMSO-d_6.

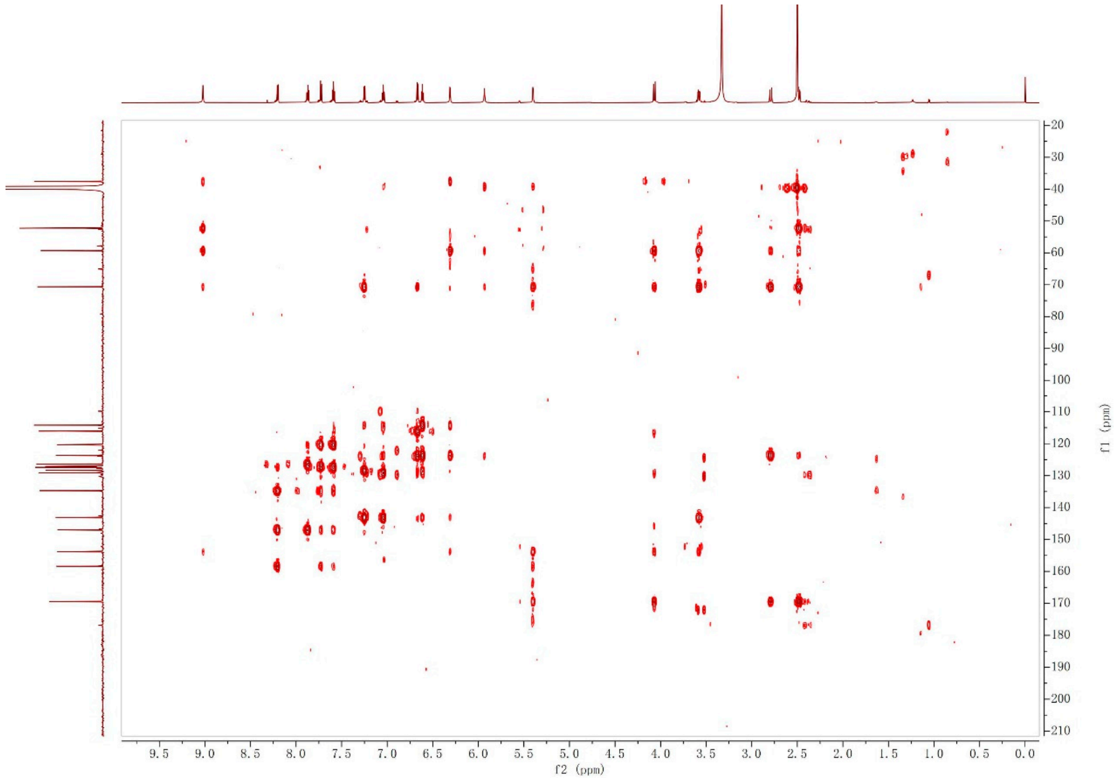

Figure S27. The HMBC spectrum of **4** in DMSO-d_6.

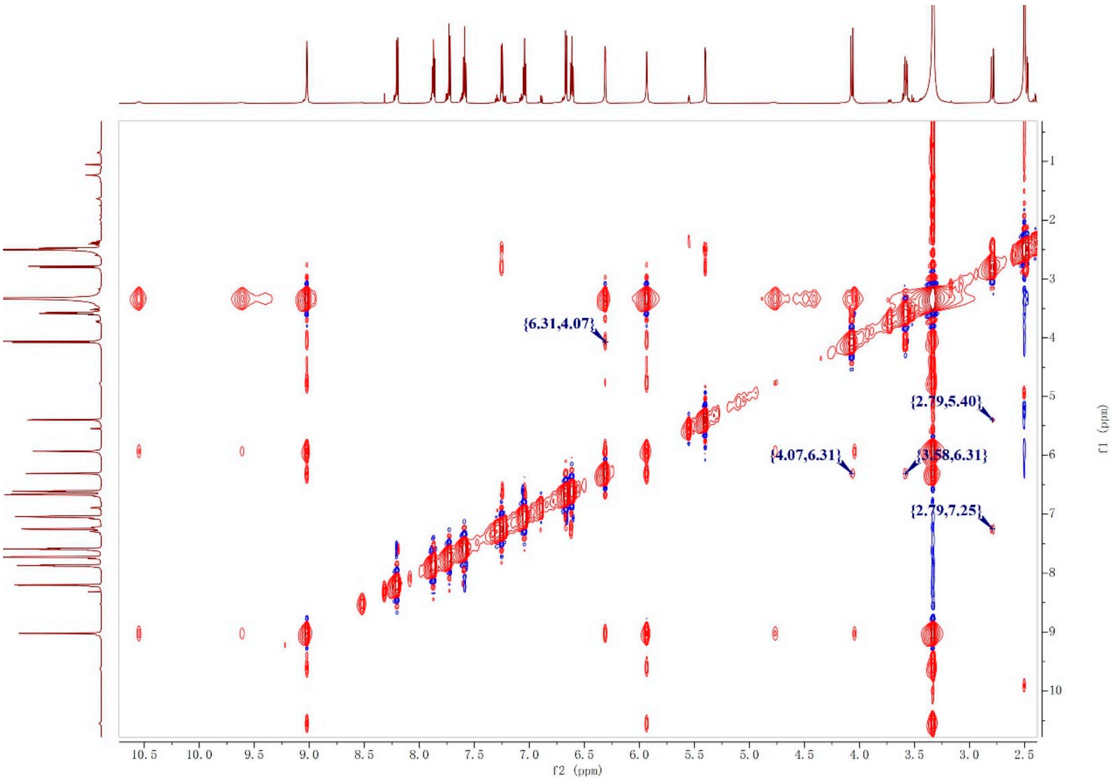

Figure S28. The NOESY spectrum of **4** in DMSO-d_6.

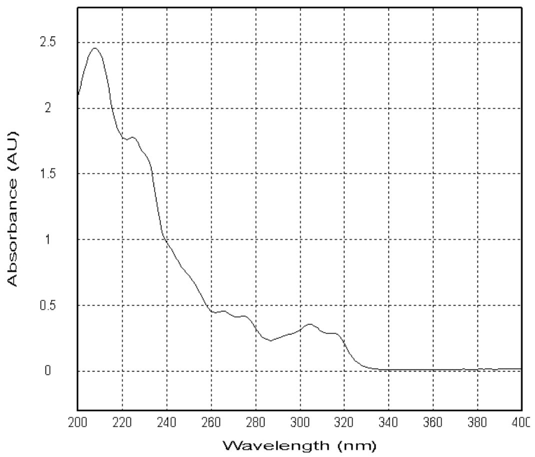

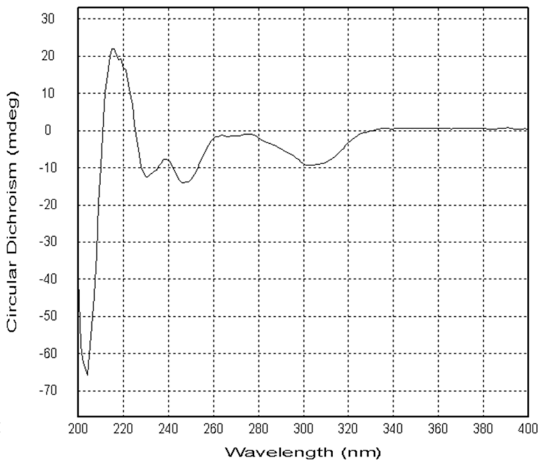

Figure S29. The UV (left) and ECD (right) spectra of **4** in MeOH.

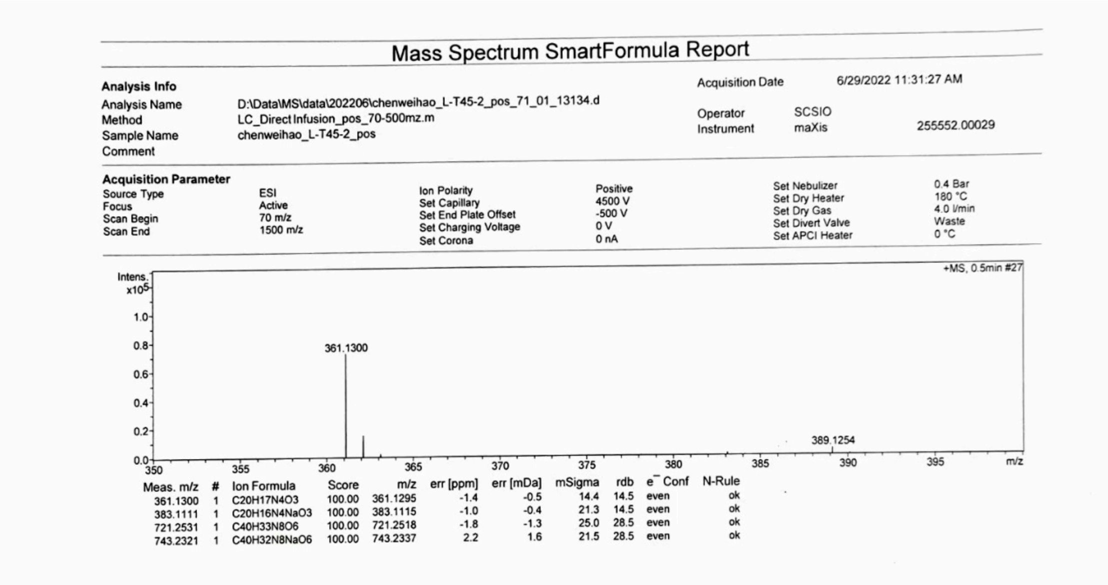

Mass Spectrum SmartFormula Report

Analysis Info

Analysis Name	D:\Data\MS\data\202206\chenweihao_L-T45-2_pos_71_01_13134.d	Acquisition Date	6/29/2022 11:31:27 AM
Method	LC_Direct Infusion_pos_70-500mz.m	Operator	SCSIO
Sample Name	chenweihao_L-T45-2_pos	Instrument	maXis 255552.00029
Comment			

Acquisition Parameter

Source Type	ESI	Ion Polarity	Positive	Set Nebulizer	0.4 Bar
Focus	Active	Set Capillary	4500 V	Set Dry Heater	180 °C
Scan Begin	70 m/z	Set End Plate Offset	-500 V	Set Dry Gas	4.0 l/min
Scan End	1500 m/z	Set Charging Voltage	0 V	Set Divert Valve	Waste
		Set Corona	0 nA	Set APCI Heater	0 °C

Meas. m/z	#	Ion Formula	Score	m/z	err [ppm]	err [mDa]	mSigma	rdb	e⁻ Conf	N-Rule
361.1300	1	C20H17N4O3	100.00	361.1295	-1.4	-0.5	14.4	14.5	even	ok
383.1111	1	C20H16N4NaO3	100.00	383.1115	-1.0	-0.4	21.3	14.5	even	ok
721.2531	1	C40H33N8O6	100.00	721.2518	-1.8	-1.3	25.0	28.5	even	ok
743.2321	1	C40H32N8NaO6	100.00	743.2337	2.2	1.6	21.5	28.5	even	ok

Figure S30. The HRESIMS spectrum of **4**.

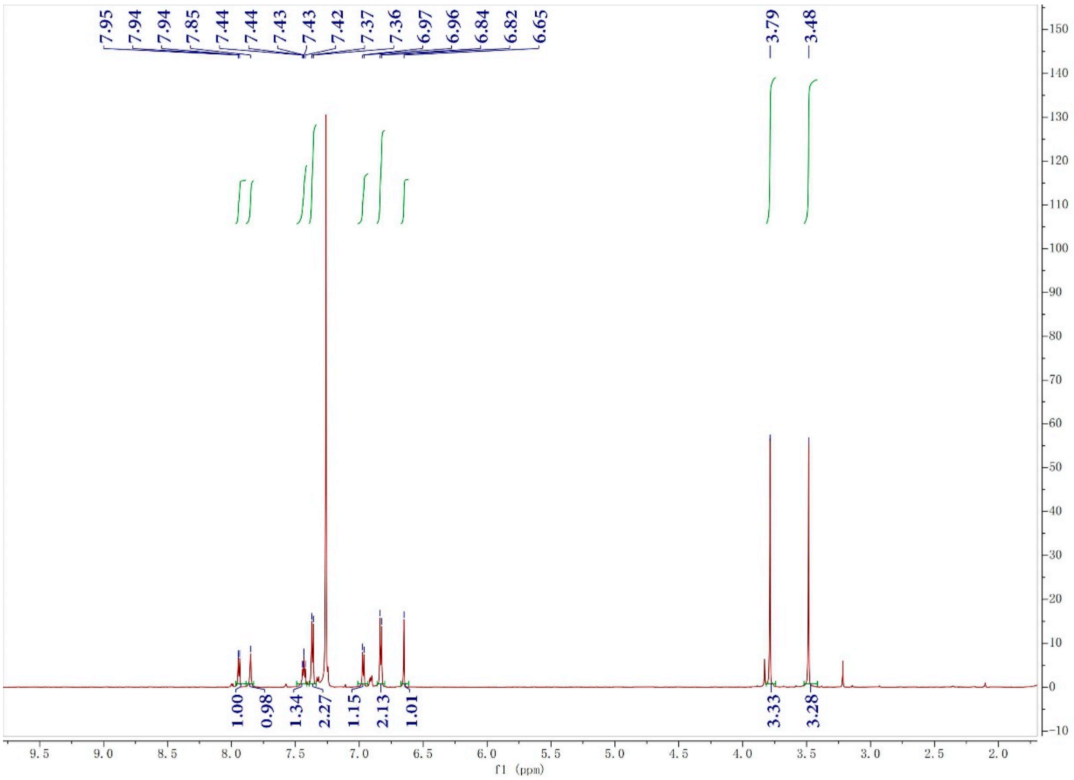

Figure S31. The ^{1}H NMR spectrum of **5** in $CDCl_3$.

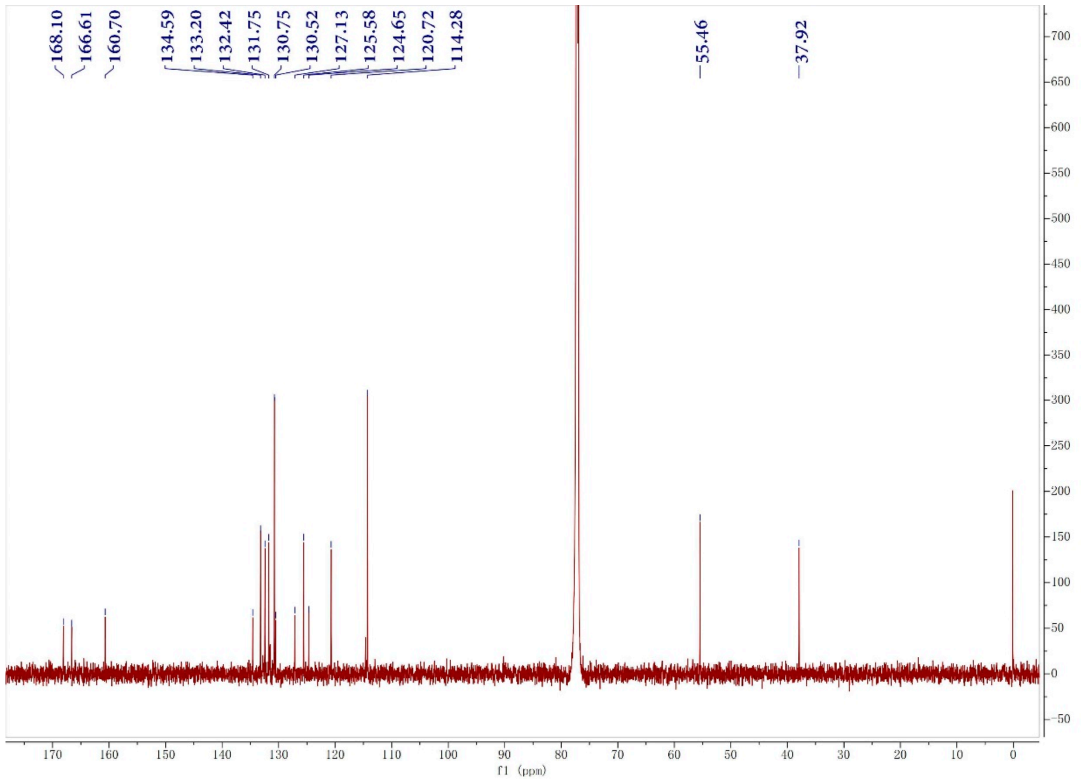

Figure S32. The ^{13}C NMR spectrum of **5** in $CDCl_3$.

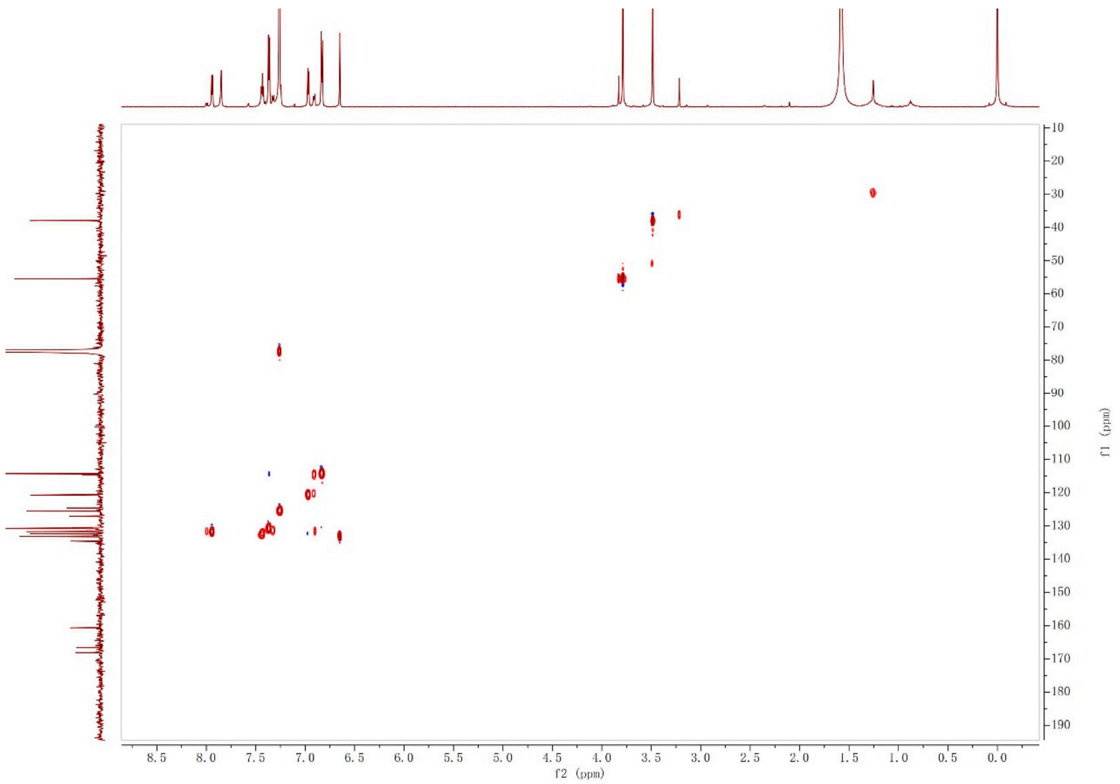

Figure S33. The HSQC spectrum of **5** in $CDCl_3$.

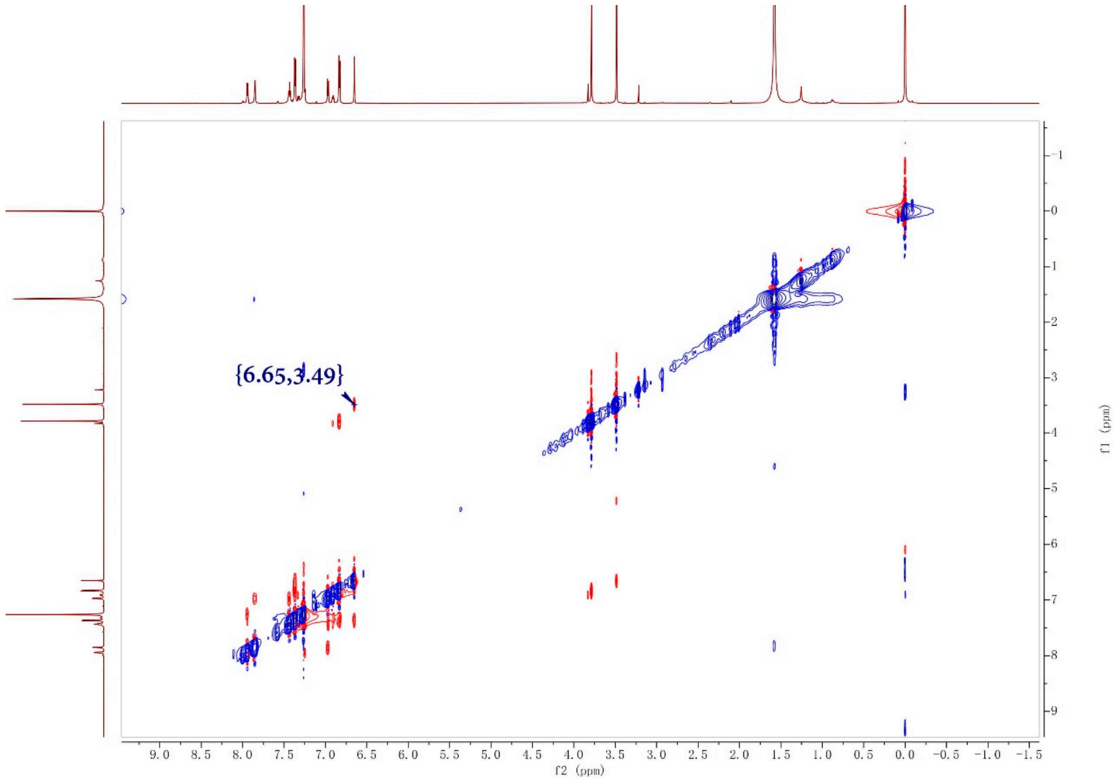

Figure S34. The NOESY spectrum of **5** in $CDCl_3$.

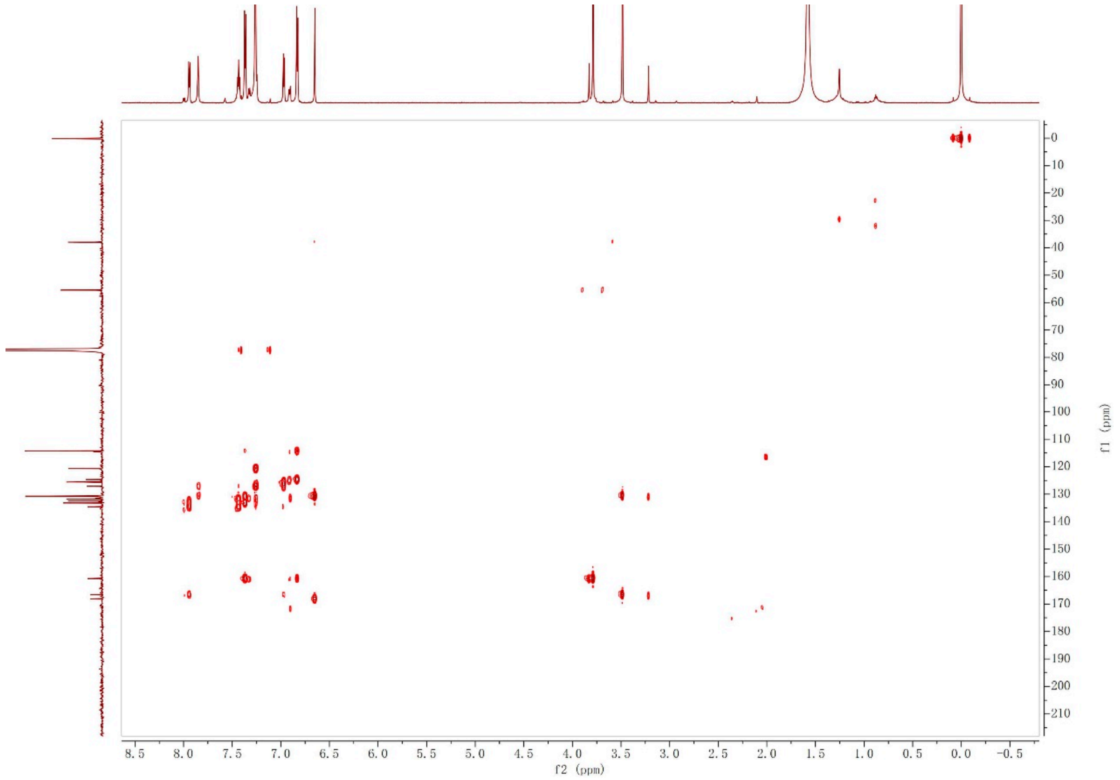

Figure S35. The HMBC spectrum of **5** in $CDCl_3$.

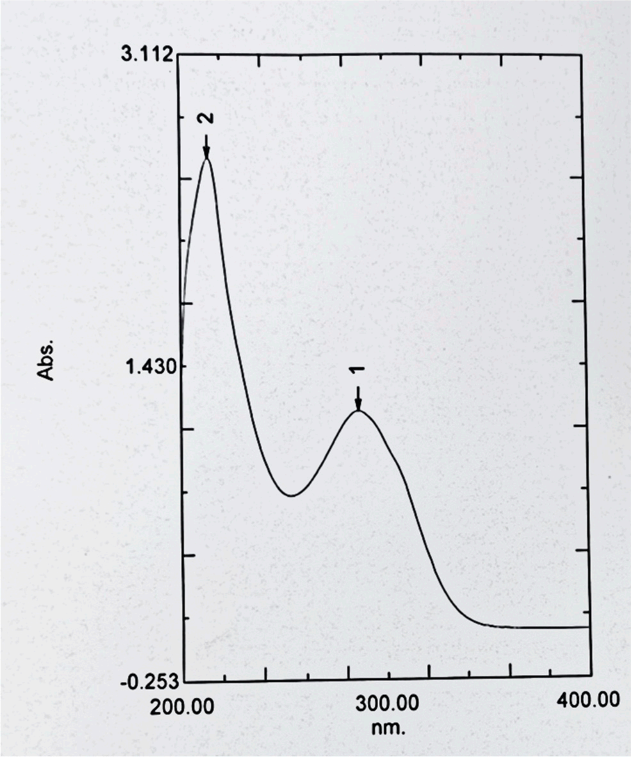

Figure S36. The UV spectrum of **5** in MeOH.

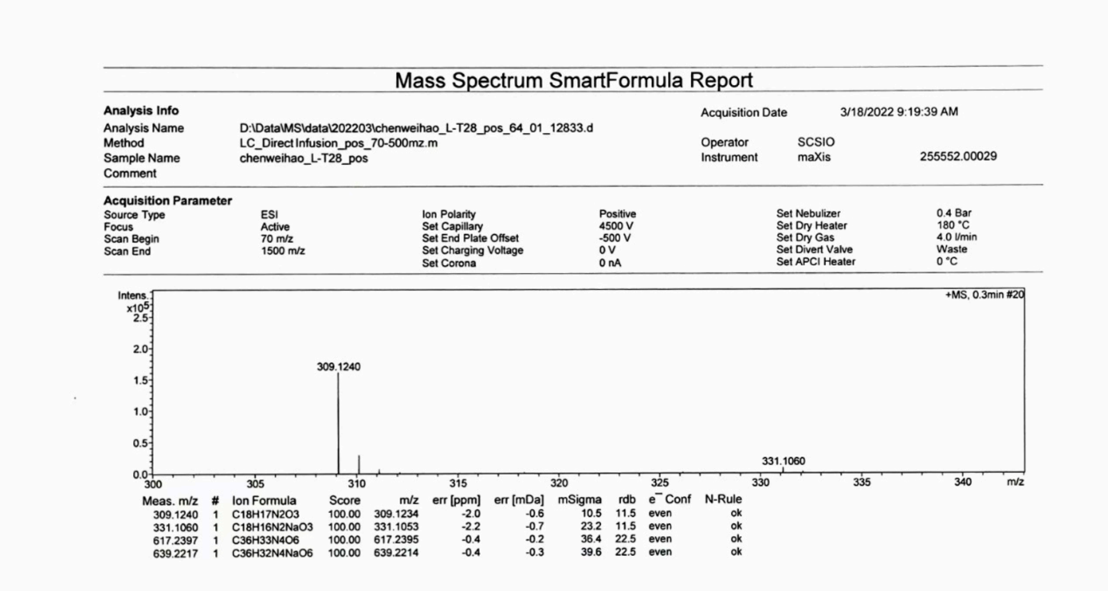

Mass Spectrum SmartFormula Report

Analysis Info

Analysis Name	D:\Data\MS\data\202203\chenweihao_L-T28_pos_64_01_12833.d	Acquisition Date	3/18/2022 9:19:39 AM
Method	LC_Direct Infusion_pos_70-500mz.m	Operator	SCSIO
Sample Name	chenweihao_L-T28_pos	Instrument	maXis 255552.00029
Comment			

Acquisition Parameter

Source Type	ESI	Ion Polarity	Positive	Set Nebulizer	0.4 Bar
Focus	Active	Set Capillary	4500 V	Set Dry Heater	180 °C
Scan Begin	70 m/z	Set End Plate Offset	-500 V	Set Dry Gas	4.0 l/min
Scan End	1500 m/z	Set Charging Voltage	0 V	Set Divert Valve	Waste
		Set Corona	0 nA	Set APCI Heater	0 °C

Meas. m/z	#	Ion Formula	Score	m/z	err [ppm]	err [mDa]	mSigma	rdb	e⁻ Conf	N-Rule
309.1240	1	C18H17N2O3	100.00	309.1234	-2.0	-0.6	10.5	11.5	even	ok
331.1060	1	C18H16N2NaO3	100.00	331.1053	-2.2	-0.7	23.2	11.5	even	ok
617.2397	1	C36H33N4O6	100.00	617.2395	-0.4	-0.2	36.4	22.5	even	ok
639.2217	1	C36H32N4NaO6	100.00	639.2214	-0.4	-0.3	39.6	22.5	even	ok

Figure S37. The HRESIMS spectrum of **5**.

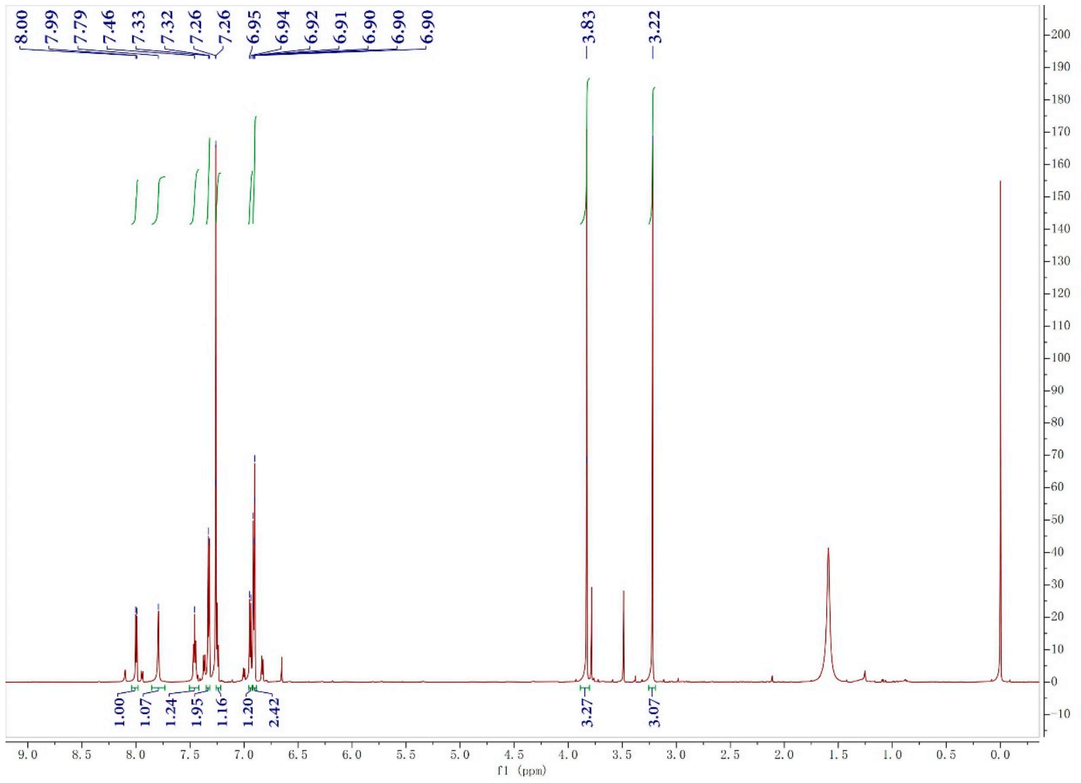

Figure S38. The ^{1}H NMR spectrum of **6** in CDCl3.

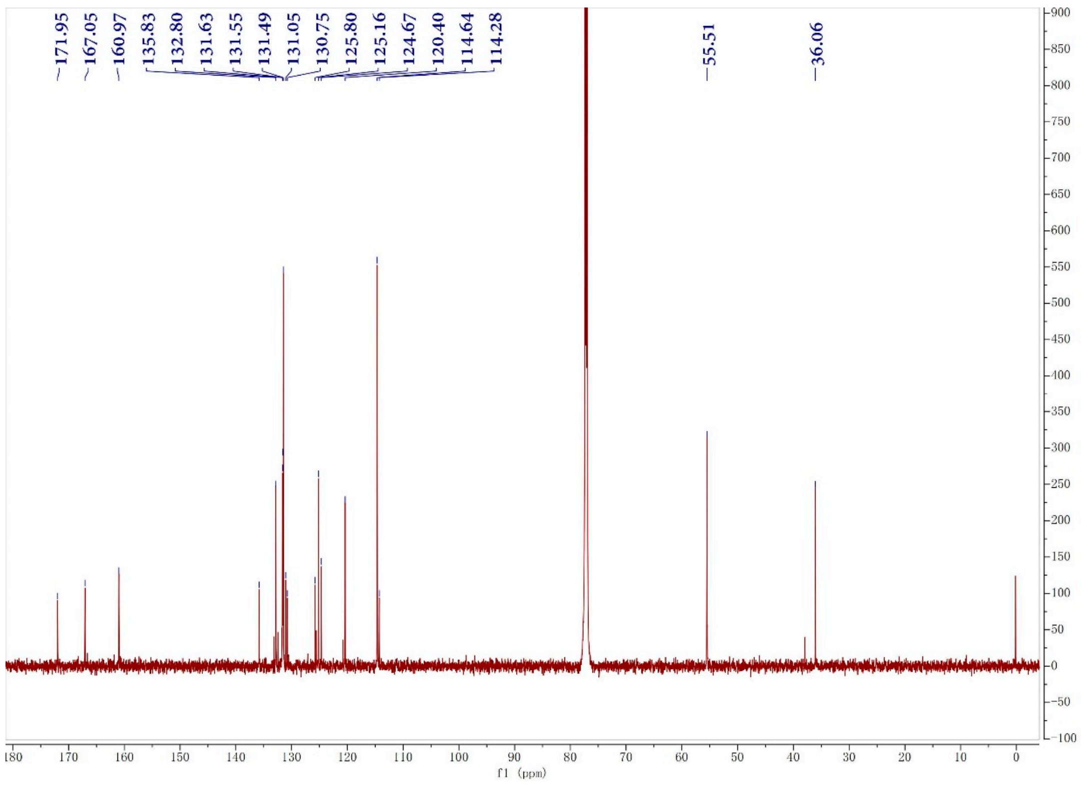

Figure S39. The ^{13}C NMR spectrum of **6** in CDCl3.

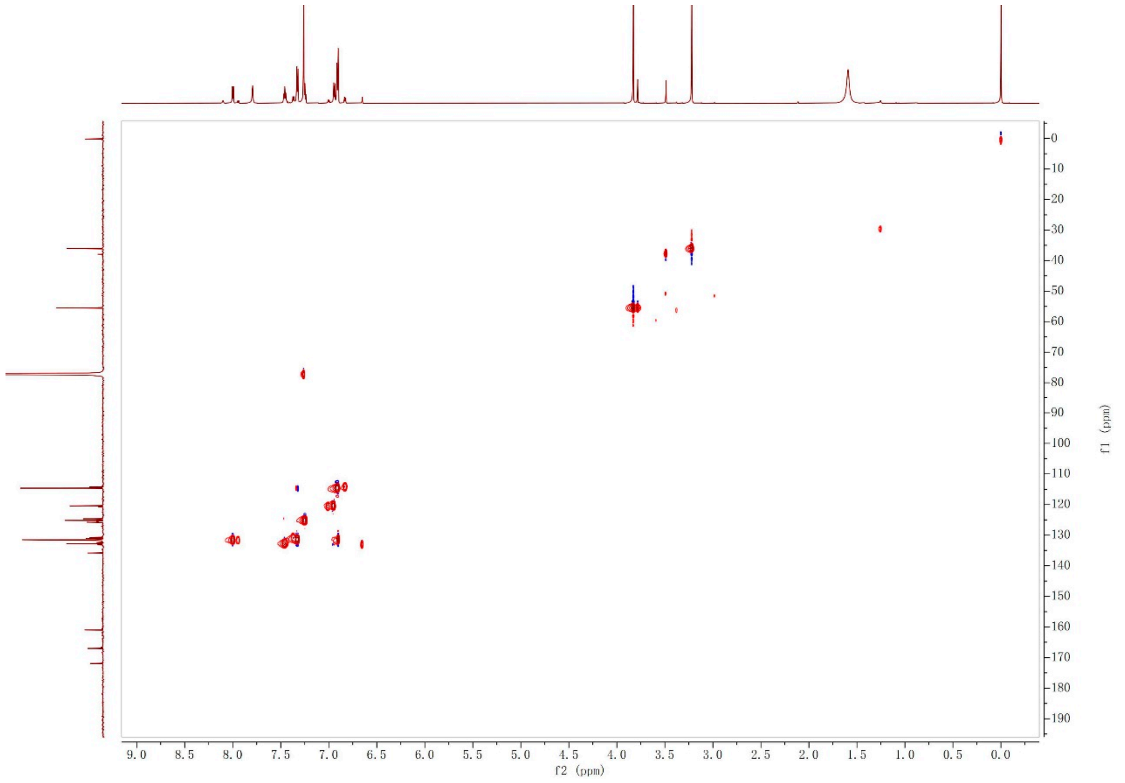

Figure S40. The HSQC spectrum of **6** in $CDCl_3$.

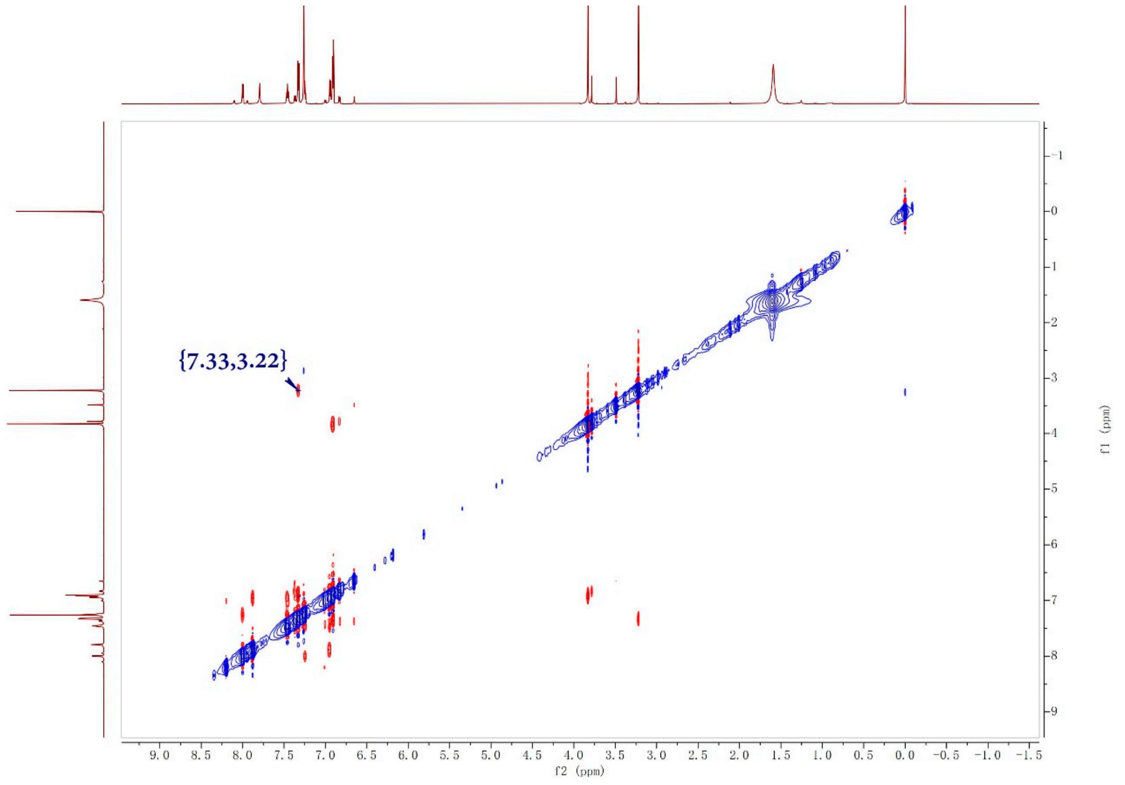

Figure S41. The NOESY spectrum of **6** in $CDCl_3$.

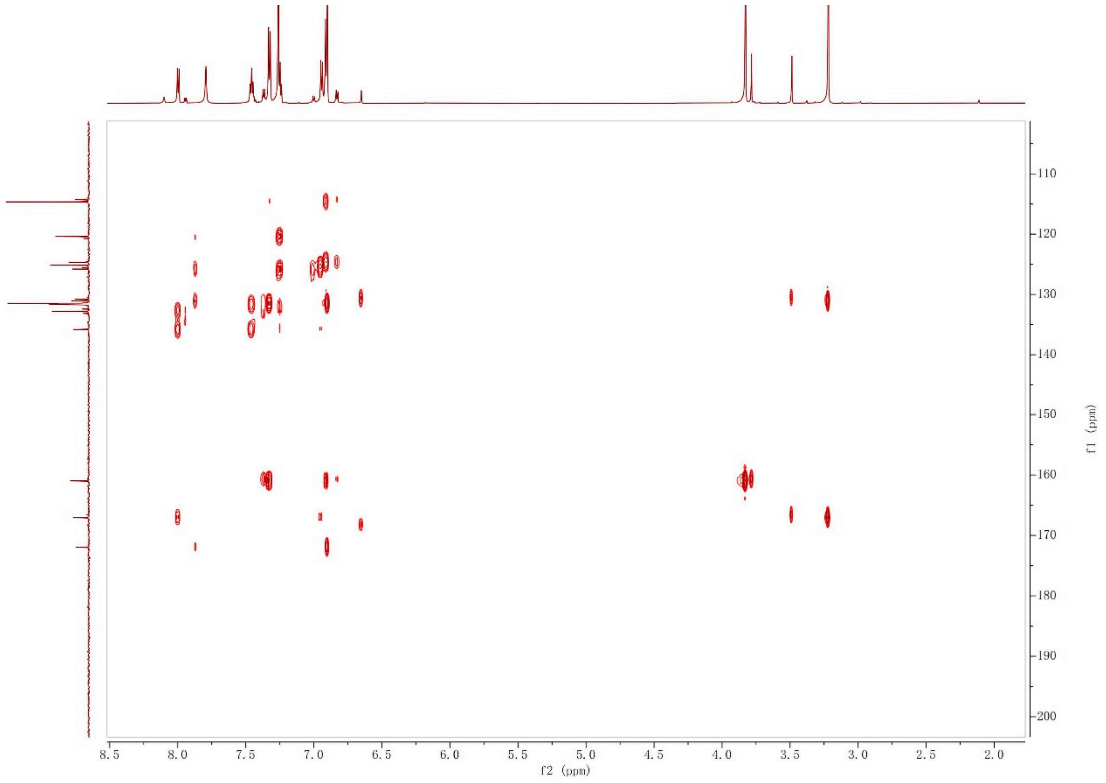

Figure S42. The HMBC spectrum of **6** in $CDCl_3$.

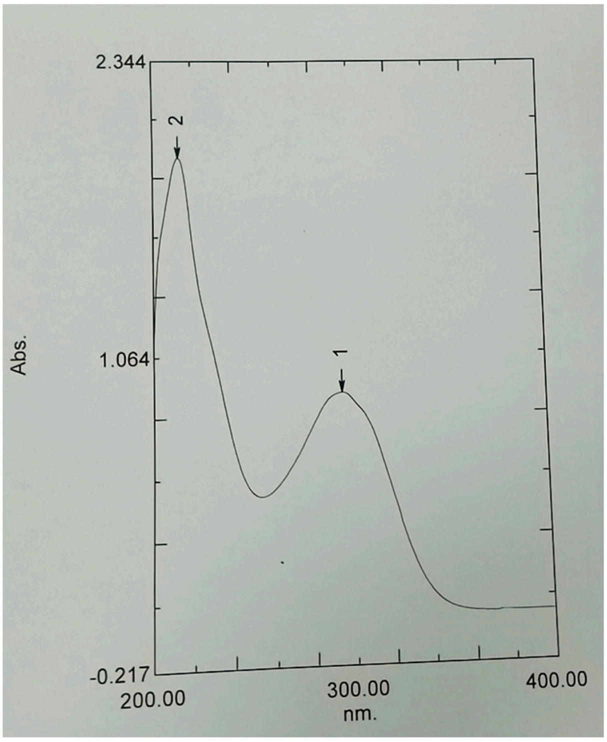

Figure S43. The UV spectrum of **6** in MeOH.

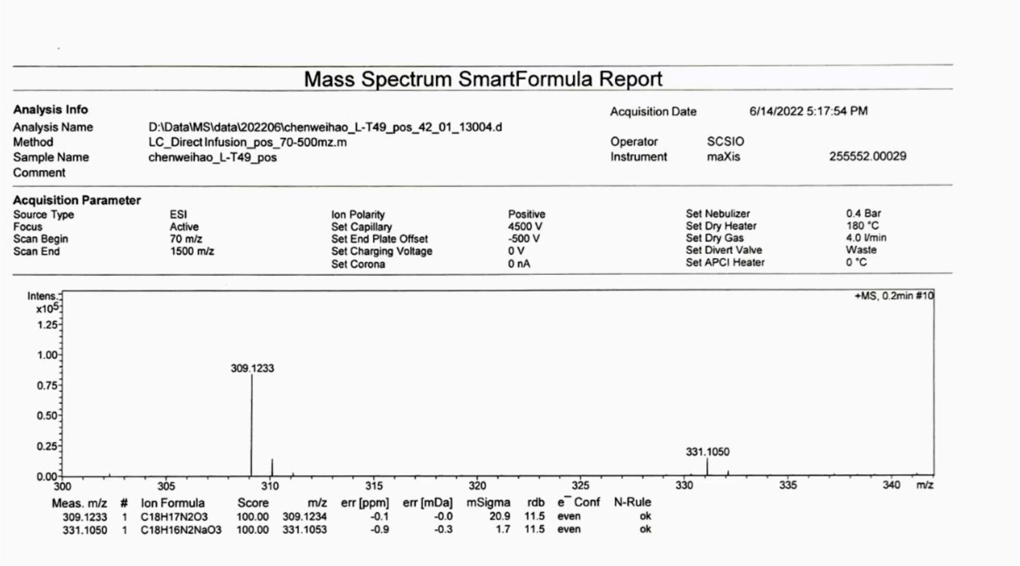

Figure S44. The HRESIMS spectrum of **6**.

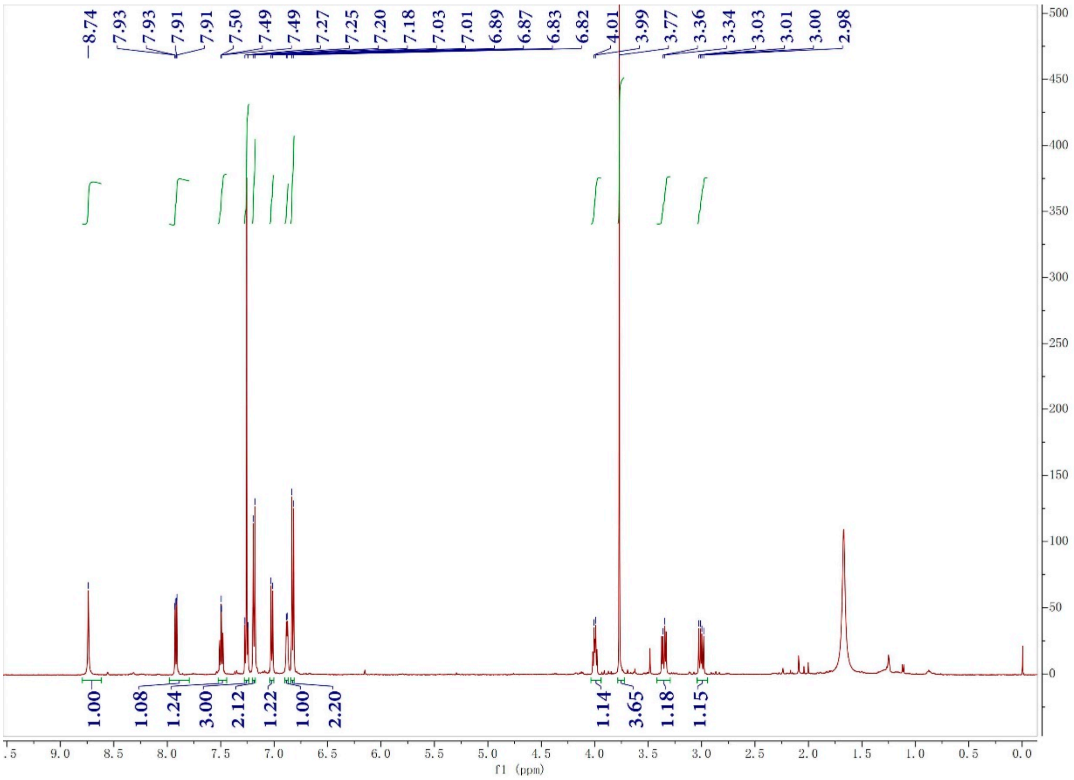

Figure S45. The ^{1}H NMR spectrum of **7** in $CDCl_3$.

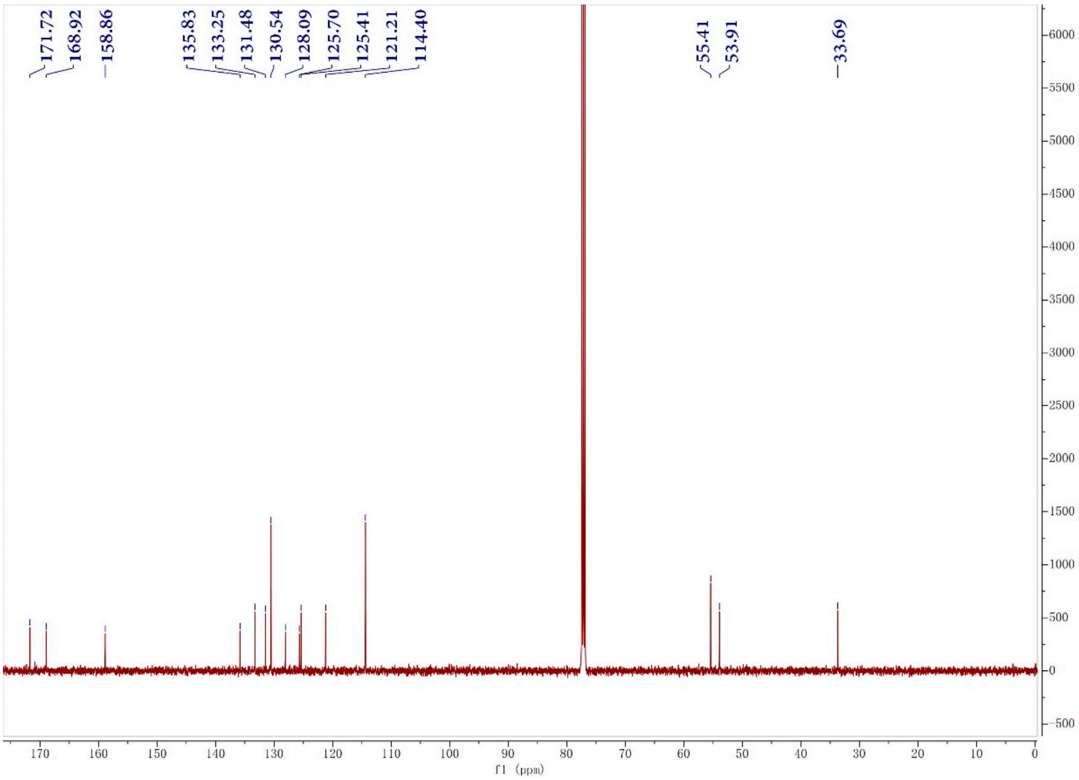

Figure S46. The ^{13}C NMR spectrum of **7** in $CDCl_3$.

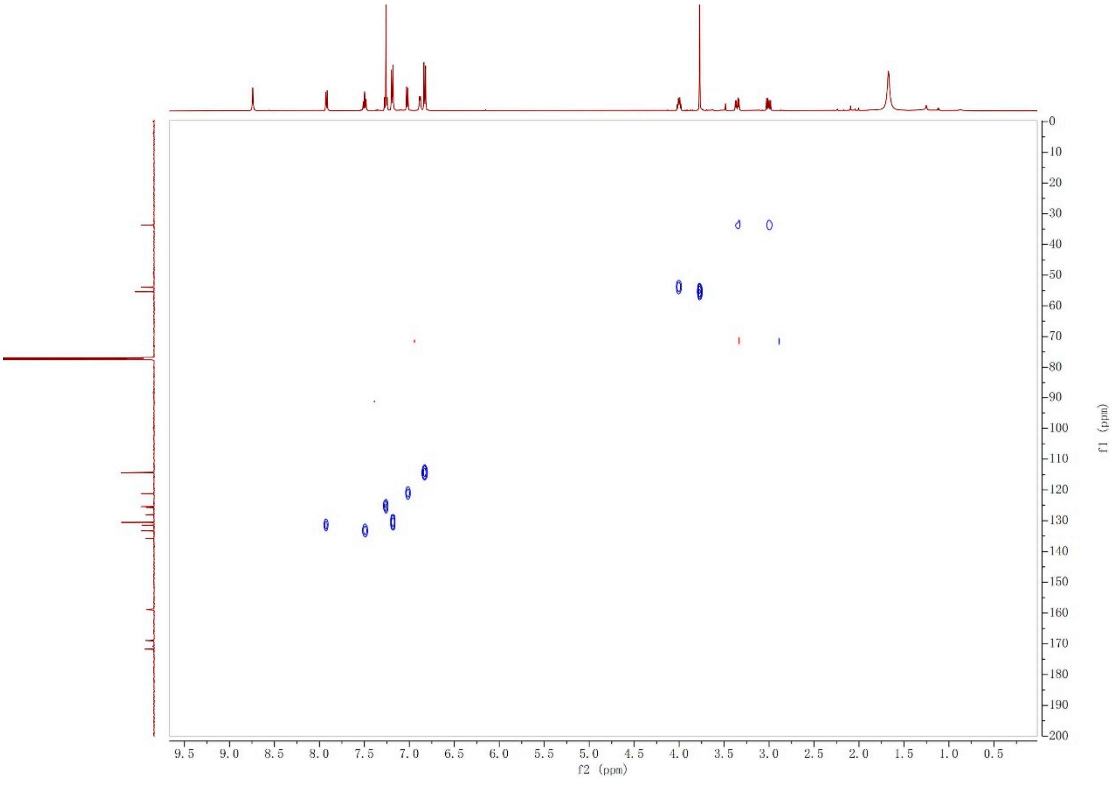

Figure S47. The HSQC spectrum of **7** in $CDCl_3$.

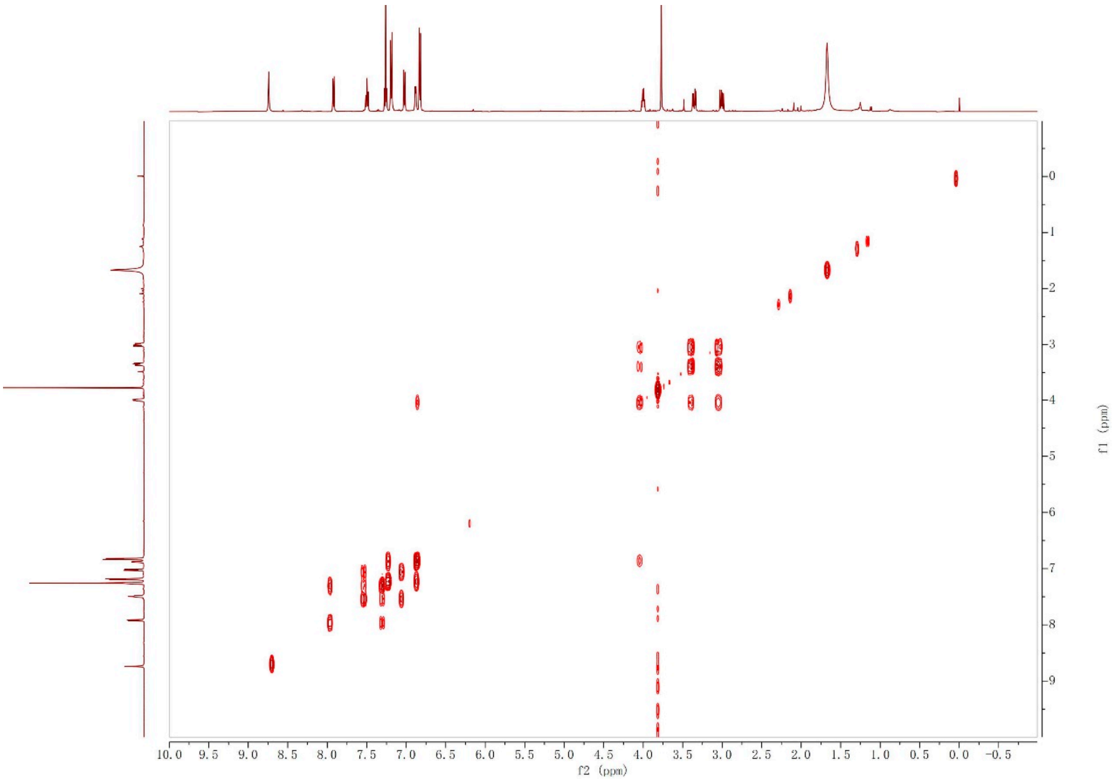

Figure S48. The ^1H-^1H COSY spectrum of **7** in $CDCl_3$.

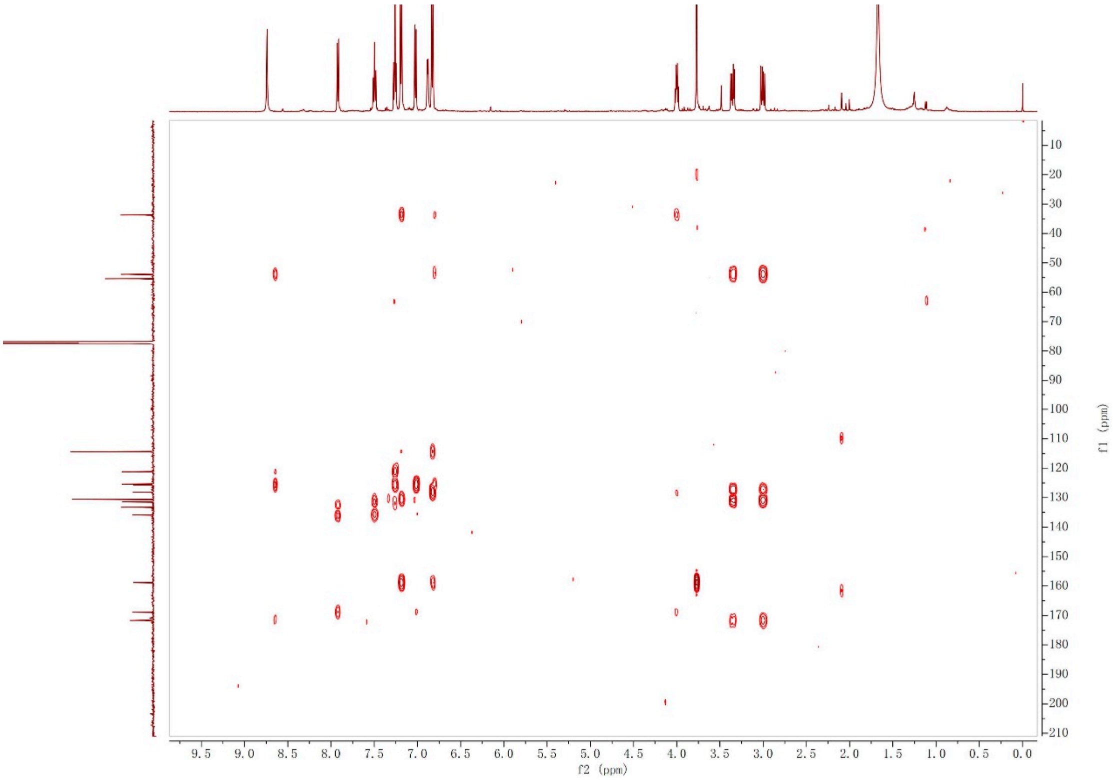

Figure S49. The HMBC spectrum of **7** in $CDCl_3$.

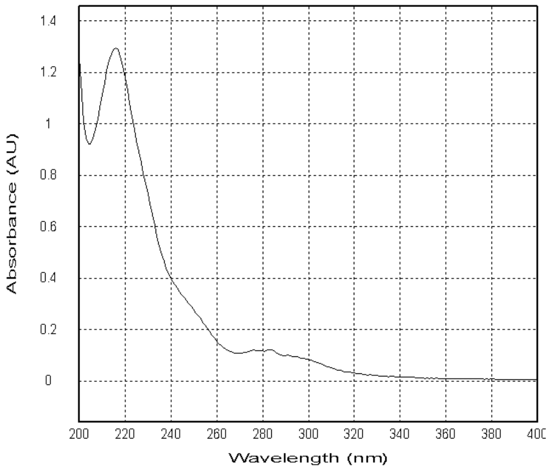

Figure S50. The UV (left) and ECD (right) spectra of **7** in MeOH.

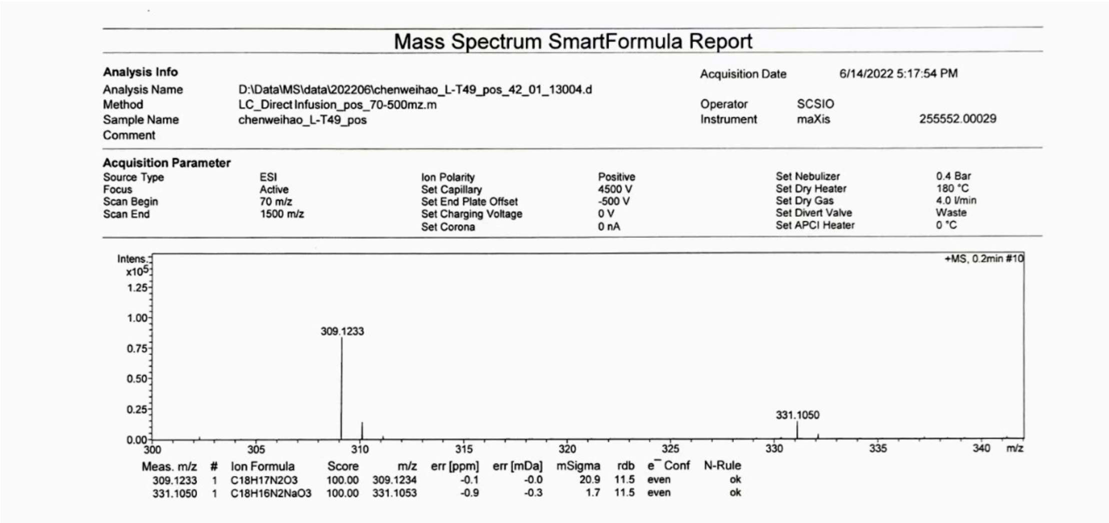

Figure S51. The HRESIMS spectrum of **7**.

Table S1. Energies of all calculated conformers at B3LYP/6-31G* level in vacuum or chloroform.

Configurations	Conformers	E (Hartree)	*Δ*E (kcal/mol)	Population (%)
**1a**	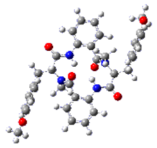	-2062.390128	0.00	86.62

**1b**	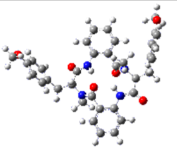	-2062.387702	1.52	6.63
**1c**	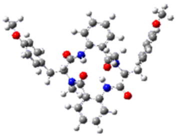	-2062.387594	1.59	5.92
**1d**	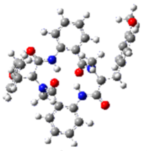	-2062.384856	3.31	0.33
**1e**	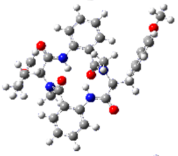	-2062.384772	3.36	0.30
**1f**	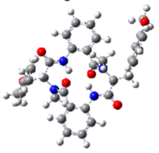	-2062.384412	3.69	0.20
**1A**	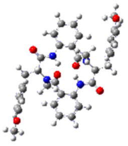	-2063.533021	0.00	64.34
**1B**	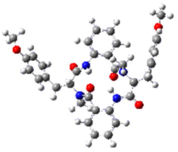	-2063.532057	0.60	23.18
**1C**	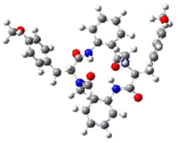	-2063.531289	1.09	10.28
**1D**	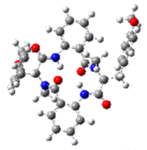	-2063.529173	2.41	1.09
**1E**	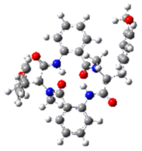	-2063.528878	2.60	0.80
**1F**	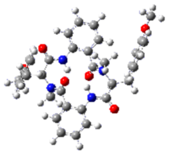	-2063.528004	3.15	0.32
**2a**	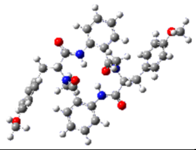	-2062.392349	0.00	56.62

**2b**	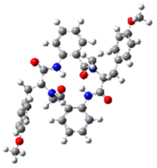	-2062.392013	0.21	39.67
**2c**	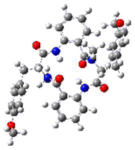	-2062.389123	2.02	1.86
**2d**	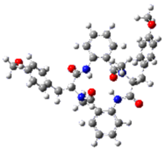	-2062.388807	2.22	1.33
**2e**	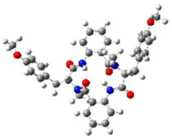	-2062.387413	3.10	0.30
**2f**	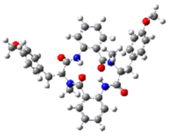	-2062.387092	3.30	0.22
**2A**	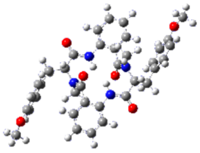	-2063.535944	0.00	46.40
**2B**	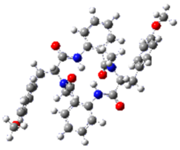	-2063.535282	0.42	23.01
**2C**	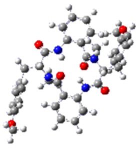	-2063.534773	0.73	13.42
**2D**	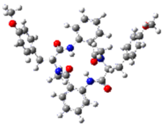	-2063.534510	0.90	10.16
**2E**	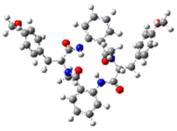	-2063.534133	1.14	6.82
**2F**	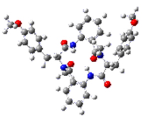	-2063.530743	3.26	0.19
